# In-depth analysis of lupeol: delving into the diverse pharmacological profile

**DOI:** 10.3389/fphar.2024.1461478

**Published:** 2024-11-13

**Authors:** Aminah Dalimunthe, Mega Carensia Gunawan, Zahirah Dhiya Utari, Muhammad Riza Dinata, Princella Halim, Nathasya Estherina S. Pakpahan, Alex Insandus Sitohang, M. Andriansyah Sukarno, Yuandani, Yahdiana Harahap, Erna Prawita Setyowati, Moon Nyeo Park, Syaratul Dalina Yusoff, Satirah Zainalabidin, Arya Tjipta Prananda, Mohd Kaisan Mahadi, Bonglee Kim, Urip Harahap, Rony Abdi Syahputra

**Affiliations:** ^1^ Department of Pharmacology, Faculty of Pharmacy, Universitas Sumatera Utara, Medan, Indonesia; ^2^ Faculty of Pharmacy, Universitas Indonesia, Jakarta, Indonesia; ^3^ Faculty of Pharmacy, Universitas Gadjah Mada, Yogyakarta, Indonesia; ^4^ Department of Internal Medicine, College of Korean Medicine, Kyung Hee University, Seoul, Republic of Korea; ^5^ College of Korean Medicine, Kyung Hee University, Seoul, Republic of Korea; ^6^ Centre for Drug and Herbal Development, Faculty of Pharmacy, Universiti Kebangsaan Malaysia, Kuala Lumpur, Malaysia; ^7^ Biomedical Science, Centre of Toxicology and Health Risk Study, Faculty of Health Sciences, Universiti Kebangsaan Malaysia, Kuala Lumpur, Malaysia; ^8^ Faculty of Medicine, Universitas Sumatera Utara, Medan, Sumatera Utara, Indonesia

**Keywords:** lupeol, triterpenoids, pharmacological properties, bioavailability, nanotechnology

## Abstract

Lupeol, a naturally occurring lupane-type pentacyclic triterpenoid, is widely distributed in various edible vegetables, fruits, and medicinal plants. Notably, it is found in high concentrations in plants like *Tamarindus indica*, *Allanblackia monticola*, and *Emblica officinalis*, among others. Quantitative studies have highlighted its presence in Elm bark, Olive fruit, Aloe leaf, Ginseng oil, Mango pulp, and Japanese Pear bark. This compound is synthesized from squalene through the mevalonate pathway and can also be synthetically produced in the lab, addressing challenges in natural product synthesis. Over the past four decades, extensive research has demonstrated lupeol’s multifaceted pharmacological properties, including anti-inflammatory, antioxidant, anticancer, and antibacterial effects. Despite its significant therapeutic potential, clinical applications of lupeol have been limited by its poor water solubility and bioavailability. Recent advancements have focused on nano-based delivery systems to enhance its bioavailability, and the development of various lupeol derivatives has further amplified its bioactivity. This review provides a comprehensive overview of the latest advancements in understanding the pharmacological benefits of lupeol. It also discusses innovative strategies to improve its bioavailability, thereby enhancing its clinical efficacy. The aim is to consolidate current knowledge and stimulate further research into the therapeutic potential of lupeol and its derivatives.

## 1 Introduction to lupeol

Triterpenoids are a major class of lipid organic compounds that play important structural and functional roles in plants, animals, and other organisms. As an essential component of the human diet, triterpenoids, primarily obtained from vegetable oils, cereals, and fruits, offer numerous health benefits (Othman and Moghadasian, 2011).

Lupeol is widespread across various species within the plant kingdom. Numerous edible vegetables and fruits, such as pepper, tomato, bitter root, white cabbage, soybean, cucumber, ivy gourd, strawberries, red grapes, carrot, pea, black tea, figs, mulberries, guava, and date palm, contain lupeol. Additionally, lupeol is abundant in medicinal plants such as *Tamarindus indica, Allanblackia monticola, Crataeva nurvala, Aegle marmelos, Bombax ceiba, Zanthoxylum riedelianum,* Shea butter plant*, Leptadenia hastata, Celastrus paniculatus, Sebastiania adenophora, Himatanthus sucuuba, Emblica officinalis,* and licorice. Studies quantifying its presence have demonstrated lupeol in the elm plant (800 μg/g bark), olive fruit (3 μg/g), aloe leaf (280 μg/g dry leaf), ginseng oil (15.2 mg/100 g of oil), mango fruit (1.80 μg/g pulp), and Japanese pear (175 μg/g twig bark) (Siddique and Saleem, 2011).

**FIGURE 1:**
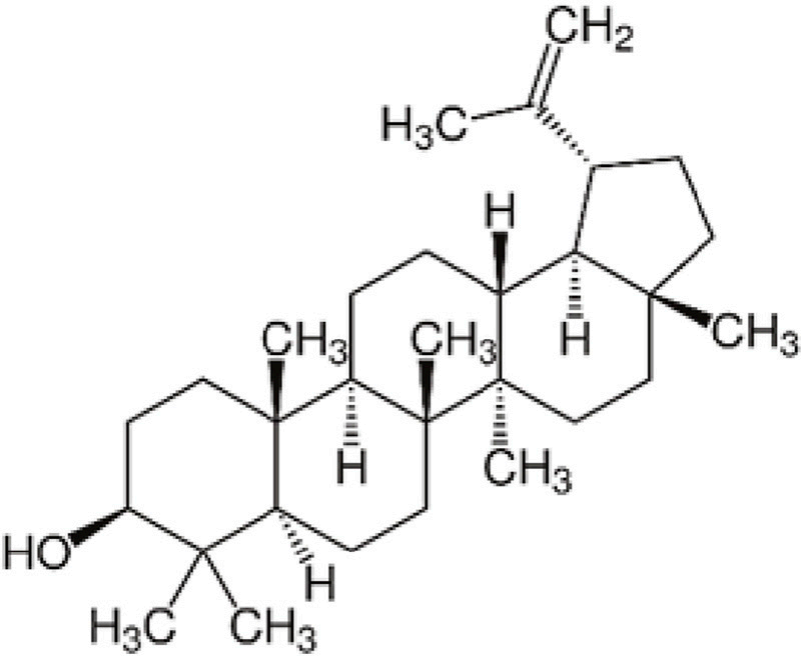
Chemical structure of lupeol.

Triterpenoids, which are derived from isopentenyl pyrophosphate oligomers, constitute the largest class of phytochemicals (Bishayee et al., 2011). Lupeol, a lupane-type pentacyclic triterpenoid, is naturally synthesized from squalene through the mevalonate pathway and is found in many vegetables and fruits. Additionally, lupeol can be synthetically produced from (S)-epoxyacetate in the lab, providing a solution to a longstanding challenge in natural product synthesis (Sohag et al., 2022). The chemical structure of lupeol can be seen on Figure 1.

Research spanning the past four decades has revealed that lupeol showcases a range of pharmacological properties such as anti-inflammatory, antioxidant, anticancer, and antibacterial effects (Liu K. et al., 2021a). Despite its significant health potential, lupeol’s clinical use has been hindered by its poor water solubility and limited bioavailability. Recent studies have used nano-based approaches to enhance lupeol’s bioavailability. Additionally, various lupeol derivatives have been created to overcome these limitations and further boost its bioactivity (Sohag et al., 2022). A recent review highlighted the pharmacological effects of lupeol and its triterpene derivatives (Sharma et al., 2020). This review explores the latest advancements concerning the pharmacological advantages of lupeol, as well as strategies to enhance its bioavailability.

## 2 Chemical characterization of lupeol

Lupeol (3-beta)-Lup-20(29)-en-3-ol (Figure 1) and other triterpenes are secondary plant metabolites that interact with their environment, particularly after infection or external damage (Chappell, 2002). The biosynthesis process of lupeol is one of nature’s most complex events, coordinated by triterpene synthases (Phillips et al., 2006; Thimmappa et al., 2014). Lupeol’s basic biosynthetic pathway is well understood because it is composed of five six-membered rings (ursanes and lanostanes) or four six-membered rings and one five-membered ring (lupanes and hopanes).

Lupeol, a triterpene, has the chemical structure (3-beta)-Lup-20(29)-en-3-ol. Its chemical formula is C_30_H_50_O, and it has a melting point of 215°C–216°C with a molecular weight of 426.7174 g/mol. In its infrared spectrum, lupeol displays a hydroxyl group and an olefinic moiety at 3,235 and 1,640 cm^−1^, respectively. Lupeol’s 1H NMR spectrum shows that it has seven methyl singlets and an olefinic function, which is typical of triterpenes. High-performance liquid chromatography (HPLC) and mass spectrometry (MS) analyses indicate that lupeol shows a parent ion peak at m/z 409 [M + H—18][+] (Sharma et al., 2020).

Lupeol is a triterpenoid, a bioactive substance found in various over-the-counter drugs. According to reports, lupeol has strong pharmacological properties, including anti-inflammatory, anti-oxidant, and anti-angiogenic effects (Agarwal and Rangar, 2003). It enhances the immune system’s response to tumor growth caused by peroxidic oil. Additionally, it maintains vital lipid profile normalization, lipophilia activity, and a protective effect against hypercholesterolemia linked to gastric damage and tubule immune factor (Gurupriya et al., 2018).

**FIGURE 2:**
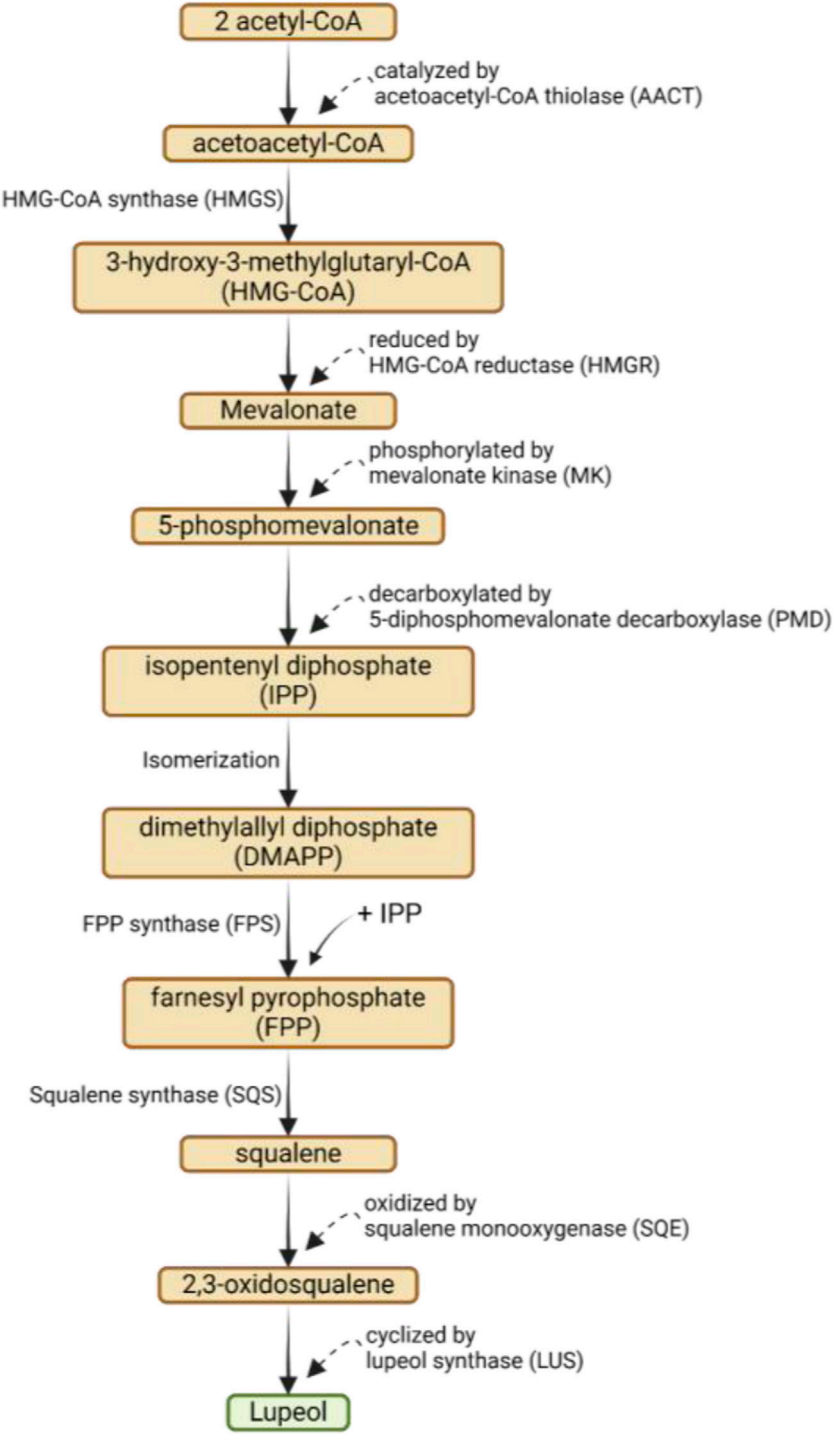
Biosynthesis of lupeol via mevalonic acid (MVA) pathway.

The biosynthesis of lupeol involves a complex series of enzymatic reactions that convert acetyl-CoA into lupeol via the mevalonic acid (MVA) pathway. The process begins with the condensation of two acetyl-CoA molecules to form acetoacetyl-CoA, catalyzed by acetoacetyl-CoA thiolase (AACT). Next, acetoacetyl-CoA is converted into 3-hydroxy-3-methylglutaryl-CoA (HMG-CoA) by HMG-CoA synthase (HMGS), which is then reduced to mevalonate by HMG-CoA reductase (HMGR). Mevalonate is phosphorylated to form 5-phosphomevalonate by mevalonate kinase (MK) and then decarboxylated to form isopentenyl diphosphate (IPP) by 5-diphosphomevalonate decarboxylase (PMD) (Bachořík and Urban, 2021; Li D. et al., 2021a). A series of isomerizations convert IPP into dimethylallyl diphosphate (DMAPP). DMAPP and another molecule of IPP combine to form farnesyl pyrophosphate (FPP) via FPP synthase (FPS). Squalene synthase (SQS) subsequently converts FPP into squalene, which is then oxidized to 2,3-oxidosqualene by squalene monooxygenase (SQE). 2,3-Oxidosqualene is cyclized into lupeol by lupeol synthase (LUS) (Bachořík and Urban, 2021; Li et al., 2021a; Liu et al., 2021b). The biosynthesis of lupeol via mevalonic acid pathway can be seen on Figure 2.

## 3 Analytical techniques for lupeol identification

Phytochemical evaluation is a method used to analyze the quality of a substance. It involves preliminary screening, chemoprofiling, and analyzing marker compounds using advanced analytical methods. The World Health Organization (WHO) introduced the use of chromatography as a method for standardizing plant products. This approach is widely regarded as a technique for identifying and evaluating the quality of plant medicines (Manjula et al., 2013). Chromatography is one of the most extensively used analytical techniques for determining the number of compounds present in each sample and identifying the chemical composition of compounds or mixtures of compounds, such as products of plant origin (Carvalho et al., 2023). Analyzing the quantity of lupeol found in natural sources is a crucial step in enhancing understanding of the characteristics associated with plant sources. Additionally, in the context of therapy, it enables the determination of the appropriate dosage of the compound to be administered and the measurement of the amount of lupeol present in new formulations. The presence of lupeol in the sample can be discovered and quantified using a combination of spectroscopic and chromatographic methods (Cruz-Salas et al., 2023). Table 1 provides an overview of the advantages and disadvantages of these techniques for lupeol determination.

**TABLE 1:** The advantages and disadvantages of different analytical techniques for determination of lupeol.

Detection method		Advantages	Disadvantages	References
Chromatographic techniques	LC	Strong qualitative ability, high sensitivity and selectivity	Not suitable to obtain material structural information	Donato et al. (2012)
	UHPLC	Greater resolution, higher sensitivity, faster analysis	Less columns life, column requires more maintenance	López-Ruiz et al. (2019)
	HPTLC	Advanced separation efficiency and detection limits, rarely require clean-up, fast quantities	Require expensive organics in big amounts, bulky equipment, large space requirement	Gupta et al. (2022)
	HPLC	Shorter time analysis, fast, automated and extremely precise technique	High cost, long detection time	Swartz (2010)
	GC	Fast, sensitive, versatile	Longer run time, low throughput, less feasibility to multi-component analysis	Cramers and Leclercq (1999)
Chromatography-coupled with spectroscopic techniques	LC-MS	High sensitivity, perform efficient separation, available for low molecular weight analytes	Expensive equipment, high cost of detection	Wu and French (2013); Grebe and Singh (2011)
	LC-APPI-MS/MS	Shorter analysis time, low total cost, low LOQ, high selectivity, good reliability	Low limits of detection that leads to decreased accuracy	Zhou et al. (2010)
	UPSFC-MS/MS	Rapid separation, high efficiency and flow rate	Lack of robustness and sensitivity	Losacco et al. (2019)
	LC-MS/MS	Specificity, sensitivity, higher accuracy	High detection cost, complex operation	Shipkova and Svinarov (2016)
	GC-MS	High resolution, high sensitivity and specificity	Longer run times, complex sample preparation of larger molecules	Majchrzak et al. (2018)
	UHPLC-MS/MS	Shorter analysis time, high specificity	Narrow analyte peaks, frictional heating effects	Wang et al. (2014b)
	HPLC-PDA	Versatile, simple, reliable, reproducible performance, relatively low cost	Low UV absorption due to lupeol structural peculiarities	Viganó et al. (2023)

One of the chromatographic methods, liquid chromatography (LC), employs buffers and additives in the mobile phases to facilitate separation. The pH of the mobile phases can be regulated to assure the ionization of the analyte, which can lead to capillary blockage, suppression of ionization, and impact the sensitivity and accuracy of the analysis, ultimately limiting the operational lifespan. When used in combination with spectroscopic techniques, LC is employed for both structural identification and confirmation. It is widely utilized in the characterization of degradation and impurities in drugs due to its remarkable precision, accuracy, specificity, selectivity, resolution, and capacity, making it a powerful tool for degrading impurity profiling (Chew et al., 2021). The measurement of lupeol in conjunction with a spectroscopic approach has been attracting interest due to the emergence of quick, sensitive, and selective ultra-high performance liquid chromatography (UHPLC). This technology enables an improvement in the efficiency of separation while significantly reducing the analytical durations (Silveira et al., 2020). Gas chromatography (GC) is another chromatographic technique that can also be applied for the analysis of lupeol compounds. But GC analysis requires longer run time, low throughput, and less feasibility than multi-component analysis as compared to other chromatographic methods. In this line, chromatography techniques coupled to mass spectrometry (MS) detectors are suitable for the characterization of complex lupeol compounds in natural products (Khatal and More, 2019).

Another alternative method for the analysis of lupeol compounds is High-Performance Thin-Layer Chromatography (HPTLC), a widely used and efficient technique for quantifying amounts and devising approaches to discover marker molecules. Currently, the HPTLC technique is extensively used for quality control of herbs and their formulations. This is because it requires a minimal amount of mobile phase and allows for the analysis of multiple samples simultaneously, leading to cost and time savings in research. It offers a comprehensive characterization of a plant extract by utilizing several wavelengths of light commonly noticed in more specialized forms of analysis. The methodology is more accurate and finely tuned and offers numerous benefits compared to other analytical methods. The increased size of stationary phases has expanded the use of HPTLC for a diverse variety of samples (Alam et al., 2018). Among the different methods available, High Performance Liquid Chromatography (HPLC) is one of the chromatographic methods that is most widely used for quantitative analysis of phytopharmaceuticals because it is able to detect more specifically in analyzing complex mixtures and has a higher level of separation. However, ultraviolet detection is limited and depends on the mobile phase used because lupeol does not have a chromophore (Oliveira et al., 2012). Further details on chromatography-coupled with spectroscopic methods for quantifying lupeol in plant sources are presented in Table 2.

**TABLE 2:** Chromatographic methods for quantification of lupeol in plant sources.

Plant sources	Analytical method			Validation parameters				References
	Chromatographic method	Mobile phase	Column	tR (min)	LOD	LOQ	Precision	
*Hygrophila schulli*	HPTLC	N-hexane: ethyl acetate (80:20, v/v)	*N/A	*N/A	20,9 ng	63,34 ng	≤2% for inter- and intra-day	Ghule et al. (2021)
*Costus igneus*	HPTLC	N-hexane: ethyl acetate (80:20, v/v)	*N/A	*N/A	131 ng	430 ng	2,43	Manjula et al. (2013)
*Vernonanthura ferruginea*	HPLC	Acetonitrile: acetic acid (99,99:0,01, v/v)	C8 reverse phase column (250 × 4.6 mm, 5 µm)	*N/A	0,38 µg	0,98 µg	*N/A	Oliveira et al. (2012)
*Coccoloba uvifera*	TLC	Acidified water (0.1% formic acid): acetonitrile	C18 (1.6 μm; 100 × 2.1 mm)	2,79	08,421 ng/mL	25,518 ng/mL	*N/A	Cruz-Salas et al. (2023)
*Jatropha gossypiifolia*	UHPLC	0,1% acetic acid in water and acetonitrile	SB-C18 Rapid Resolution HD column (2.1 × 50 mm, 1.8 μm)	6,590	*N/A	*N/A	*N/A	Silveira et al. (2020)
*Hemidesmus indicus*	HPTLC	Hexane: ethyl acetate (8:2, v/v)	C18 column (5 μm, 250 × 4.6 mm)	0,48	00,181 µg/band	00,541 µg/band	Intra day: 2,10; Inter day: 1,34	Thatipelli et al. (2023)
*Decalepis hamiltonii*	HPTLC	Hexane: ethyl acetate (8:2, v/v)	C18 column (5 μm, 250 × 4.6 mm)	0,48	00,181 µg/band	00,541 µg/band	Intra day: 2,10; Inter day: 1,34	Thatipelli et al. (2023)
*L. reticulata*	HPTLC	Petroleum ether: ethyl acetate: acetonitrile (8.2:1.2:0.1, v/v/v)	*N/A	*N/A	0,03 μg/band	0,10 μg/band	ND*	Mallick and Dighe (2014)
*Betula utilis*	HPTLC	N-hexane: ethyl acetate (8:2, v/v)	*N/A	*N/A	60 ng/band	80 ng/band	Intra day: 1,044; Inter day: 693,27	Khan et al. (2012)
*Tephrosia purpurea*	HPTLC	Toluene: ethyl acetate: formic acid (9:1:1, v/v/v)	*N/A	*N/A	36,01 ng/band	109,126 ng/band	*N/A	Khatoon et al. (2019)
*Vernonia cinerea*	HPLC	Methanol: acetonitrile (30:70) v/v	C18 column (150 × 4.6, 5 µm)	4,45	7 μg/mL	10 μg/mL	Intra day: 0,35%; Inter day: 0,63%	Shah et al. (2010)
*Ficus religiosa*	HPTLC	Toluene:methanol (9:1 v/v) (10 mL)	*N/A	*N/A	50 ng	100 ng	Intra day: 0,55; Inter day: 0,51	Rathee et al. (2015)
*Hygrophila auriculata*	HPTLC	Toluene: methanol-formic acid (7.0: 2.7: 0.3 v/v/v)	*N/A	*N/A	45	75	Intra day: ≤1,80%; Inter day: ≤2,18%	Hussain et al. (2012)
*Convolvulus pluricaulis*	HPTLC	Toluene: ethyl acetate: formic acid (8.5:1.5:0.1)	*N/A	*N/A	1,41 ng/band	36,01 ng/band	Intra day: 0.063-0,22; Inter day: 0.063-0,73	Irshad and Khatoon (2018)
*Hibiscus sp*	HPTLC	Chloroform: methanol (97:3; v/v)	*N/A	*N/A	31,57 ng	95,68 ng	Intra day: 1,168-1,316; Inter day: 1,160-1,312	Alam et al. (2018)
*Ficus pseudopalma*	TLC	Hexane, ethyl acetate, acetone and methanol	Reverse-phase C18 column	9,09	*N/A	*N/A	*N/A	Santiago and Mayor (2014)

Abbreviations: HPTLC (High Performance Thin Liquid Chromatography); TLC (Thin Liquid Chromatography); UHPLC (Ultra High Performance Liquid Chromatography).

*N/A: information is not provide by the original article.

Recently, advancements have been made in combining HPLC with other spectroscopic detection methods through combined approaches. The utilization of online detection and identification methods that enable chemical screening of plant extracts for various phytochemicals is a highly promising advancement in the determination and structural studies of natural products. Several investigations have employed high-performance liquid chromatography (HPLC) in conjunction with mass spectrometry (MS) to detect and characterize natural chemicals present in biological materials (Guliyev et al., 2004).

Mass spectrometry, in conjunction with a separation technique such as gas chromatography (GC), liquid chromatography (LC), or capillary electrophoresis (CE), is currently a crucial tool for the detection and characterization of tiny chemical compounds. Gas chromatography-mass spectrometry (GC-MS) is a highly effective and precise technology used to detect the lupeol chemical in medicinal plants. It provides fully resolved ion species by comparing them with existing databases. So far, a range of analytical identification approaches have been employed to ascertain the secondary metabolites present in plants. Continual progress in efficient and innovative techniques for separating substances and highly sensitive mass spectrometers with exceptional accuracy and precision presents fresh possibilities for accurately measuring secondary metabolites. A single investigation utilizing GC-MS or LC-MS/MS techniques can identify several secondary metabolites in complicated biological matrices (Ertas et al., 2015). Several research studies have employed gas chromatography-electron ionization/mass spectrometry (MS) to evaluate triterpenes. Nevertheless, silylation is essential for converting nonvolatile compounds into volatile ones. In addition, it has been noted that silylation is associated with drawbacks such as limited repeatability and instability of derivatives during examination. Research by Rhourri-Frih et al. (2009) compared three ionization sources: atmospheric pressure sources, electrospray ionization (ESI), atmospheric pressure chemical ionization (APCI), and atmospheric pressure photoionization (APPI) for the analysis of pentacyclic triterpenes, such as lupeol, to address the silylation issue and improve the sensitivity of the analysis technique.

Furthermore, researchers have proposed combining HPLC with evaporative light scattering detection (ELSD), mass spectrometry (HPLC-MS), and atmospheric pressure chemical ionization (APCI) or atmospheric pressure photoionization (APPI) to address the limitations of LC-UV. This is because UV detection occurs at wavelengths below 200 nm, making it unsuitable for determining low amounts of triterpenes like lupeol that lack chromophore groups. Therefore, gas chromatography (GC) remains a more versatile and suitable technique for the successful separation of natural components present in these plants (Jemmali et al., 2016).

However, lupeol, which is a neutral triterpenoid, has high lipophilicity and few polar functional groups, which makes it unable to be ionized and detected by mass spectrometry (MS) easily. Another factor is the possible ion suppression effect in complex matrices and relatively narrow dynamic range; for example, NMR detection systems are difficult to use due to the complexity of lupeol. The widest application is found by reversed-phase HPLC with ultraviolet (UV) or photodiode array (PDA) detection as a non-selective and universal detector. The main problem with this technique is that most triterpenoids, due to their structural peculiarities, lack chromophores and have very low UV absorption (Naumoska and Vovsk, 2015). To get better sensitivity, detection at low wavelengths (205–210 nm) is needed against strong solvent absorption. This means that the mobile phase and other chromatographic parameters can’t be chosen from a wide range of options. Nevertheless, it has advantages such as versatility, simplicity, reliability, reproducible performance, and relatively low cost (Vilkickyte and Raudone, 2021).

Recent reviews say that supercritical liquid chromatography coupled with tandem mass spectrometry SFC-MS/MS systems are ideal for separating analytes with similar structures and mass spectra, one of which is lupeol. Supercritical liquid chromatography coupled with tandem mass spectrometry SFC-MS/MS systems utilize liquid organic modifiers with different polarities, which when combined with supercritical CO2 and various stationary phases result in unparalleled versatility and selectivity. With its high flow rate and efficiency, SFC provides rapid separation. Moreover, since there is no water in the mobile phase, the interaction between the compounds and the stationary phase has a greater role in the separation compared to reversed-phase liquid chromatography, where water dominates the retention mechanism (Lesellier et al., 2012; Jayantha et al., 2023). Additional information on chromatography-coupled spectroscopic methods used for the quantification of lupeol in plant sources is provided in Table 3.

**TABLE 3:** Chromatography-coupled spectroscopic methods for quantification of lupeol in plant sources.

Plant sources	Analytical method			Validation parameters				References
	Method	Mobile phase	Column	tR (min)	LOD	LOQ	Precision	
*Alhagi maurorum*	LC-MS	Methanol and formic acid	*N/A	4	0.2 mg/L	0.9 mg/L	*N/A	Laghari et al. (2011)
*Asteracantha longifolia*	LC-ESI-MS	Water containing 2 mL/L acetic acid: acetonitrile (20:80)	Agilent Poroshell C8, 120Å, 75 mm, 4.6 mm, 2.7 µm	6.43	<0.13 ng/mL	0.4 ng/mL	≤5%	Raval et al. (2024)
*Cecropia obtusa*	LC-APPI-MS/MS	70% acetonitrile/water from 0 to 3.5 min 100% acetonitrile after 4.0 min	Zorbax SB C18 2.1 × 50 mm (1.8 μm)	*N/A	0.0020 mg/L	0.0066 mg/L	Intra day: 0.62%; Inter day: 3.43%	Gobo et al. (2016)
*Cocos Nucifera* and *Elaeis guineensis*	UPSFC-MS/MS	*N/A	C18 SB (3.0 mm × 100 mm, 1.7 µm)	1.42	10 ng/mL	20 ng/mL	*N/A	Jayantha et al. (2023)
*Madhuca longifolia*	LC-MS/MS	*N/A	C18 column (50 × 2.0 mm, 3 μm id)	3.08	2.60, μg/mL	7.90 μg/mL	Inter day: 0.9%	Patel et al. (2021)
*Malus domestica*	GC-MS	*N/A	Rtx-5ms capillary column (60 m × 0.25 mm × 0.25 μm)	19.47	*N/A	*N/A	<11%	Jemmali et al. (2016)
*Quertus obtusata*	GC-MS	Hexane:ethyl acetate (90:10)	HP-5MS capillary column (30 × 0.25 mm i.d.× 0.25 μm)	38.376	*N/A	*N/A	*N/A	Cháirez-Ramírez et al. (2015)
*Salvia hypargeia*	GC-MS	*N/A	HP-5MS (30 m × 0.25 mm × 0.25 mm)	31.94	1,314.50 μg/L	1,347.86 μg/L	0.0037%	Bakir et al. (2020)
*Syzygium cumini*	GC-MS	*N/A	Rtx-1ms (100% dimethylpolysiloxane)	*N/A	*N/A	*N/A	<2%	Chache et al. (2020)
*Taraxacum kok-saghyz*	UHPLC-MS/MS	phase A (water, 0.1% formic acid); phase B (acetonitrile-methanol (9:1, v/v)	C18 column (100 mm × 2.1 mm, 1.7 µm)	3.67	0.50 ng/g	1 ng/g	Intra day: 2.50%; Inter day: 4.54%	Kong et al. (2021)
*Vaccinium vitis-idaea*	HPLC-PDA	Acetonitrile: methanol (10:90, v/v)	ACE C18 (150 × 4.6 mm, 3 μm)	*N/A	0.14 μg/mL	0.41 μg/mL	Intra day: 0.28%; Inter day: 0.32%	Vilkickyte and Raudone (2021)

Abbreviations: APPI (Atmospheric Pressure Photoionization); ESI (Electrospray Ionization); GC (Gas Chromatography); HPLC (High Performance Liquid Chromatography); LC (Liquid Chromatography); MS (Mass Spectrometry); PDA (Photodiode Array); UHPLC (Ultra High Performance Liquid Chromatography); UPSFC (Ultra Performance Supercritical Fluid Chromatography).

*N/A: information is not provide by the original article.

## 4 Bioavailability and metabolic pathways

Lupeol is a pentacyclic triterpene that is distinguished by its diverse pharmacological effects. These effects include antioxidant, anticancer, cardioprotective, antimicrobial, antidiabetic, anti-inflammatory, anti-atherosclerotic, antimutagenic, antiproliferative, hepatoprotective, gastroprotective, renoprotective, and other effects (Gayathri et al., 2019a; Preetha et al., 2006; Tiwari et al., 2019; Cruz-Salas et al., 2023). Lupeol’s pharmacological effectiveness has been proven in some *in vivo* tests, and it has been observed to selectively target diseased and unhealthy human cells (Siddique and Saleem, 2011). In addition to its efficacy, it is regrettable that lupeol has poor bioavailability, which affects the applications of lupeol’s distribution (Malinowska et al., 2021). Lupeol is classified as a class II molecule according to the Biopharmaceutics Classification System (BCS), which means low solubility and high permeability (Liu et al., 2021a). Research studies have shown that lupeol has poor gastrointestinal absorption based on *in silico* methods. Furthermore, *in vivo* experiments have indicated that the bioavailability of lupeol through oral administration is less than 1% (Ahmed and Alkali, 2019; Liu et al., 2021a).

Lupeol’s low solubility and bioavailability come from its high lipophilicity and poor water solubility, driven by its structural characteristics. With an octanol/water partition coefficient (Log Po/w) of 7.67, lupeol’s significant lipophilicity hinders its solubility in water, reducing its absorption in aqueous environments like the gastrointestinal tract (Pardo-Rodriguez et al., 2023; Park et al., 2023). Its molecular weight of 426.72 g/mol and a low topological polar surface area (TPSA) of 20.23 further contribute to its hydrophobic nature and diminished gastrointestinal absorption. The estimated solubility (ESOL) values, with logarithms of −8.16 for α-amyrin and −9.13 for lupeol acetate, indicate poor solubility (Pardo-Rodriguez et al., 2023). Although lupeol has hydroxyl groups, they do not substantially enhance its water solubility, and esterification, such as in lupeol acetate, only marginally improves buffer solubility. This is due to the weak C-H interactions of the ester groups, which are insufficient to counterbalance the molecule’s overall hydrophobic nature, leaving its solubility in aqueous environments largely unaffected (Malinowska et al., 2019).

During the early stages after lupeol administration, the stomach exhibited the most significant accumulation of lupeol, followed by the small intestine, large intestine, liver, and ultimately the kidneys in female CD-1 mice. The distribution of lupeol is as follows: perirenal fat, ovary, spleen, mammary gland, uterus, bladder, lymph node, liver, small intestine, caecum, lung, thymus, colon, kidney, skin, heart, and brain (Udeani et al., 1999; Chaturvedi et al., 2008). One noteworthy feature is that lupeol has the ability to penetrate interior organs after crossing the intestinal barrier (Cháirez-Ramírez et al., 2019). In addition to its penetration, based on research conducted by Qin et al. (2024), it was shown that lupeol exhibits an intense affinity for the bile acid (BA) receptor, which is the Farnesoid X Receptor (FXR)—a ligand-activated nuclear protein that is expressed in the liver and gut. FXR controls bile acid production, transportation, metabolism, and reabsorption. Lupeol administration significantly lowered the protein expression of CYP7A1, increasing the diversion pathway of BA production, hence enhancing the accumulation of BA and activation of the BA feedback loop of the FXR signaling pathway. Additionally, lupeol has an effect on insulin signaling and GLUT4 protein expression (Padmapriya et al., 2019). Further research is necessary to obtain additional knowledge regarding bioavailability and metabolic pathways, as the existing information is insufficient.

## 5 Pharmacological activity of lupeol

### 5.1 Antioxidant and reactive species scavenging mechanisms

Oxidative stress is defined as an imbalance between prooxidant levels and antioxidant capacity, which contributes to the pathobiology of numerous diseases. Dietary antioxidants have been shown to prevent oxidative stress by quenching reactive oxygen species (ROS) or activating the cellular antioxidant defense system (Hannan et al., 2020). Antioxidants primarily function by eliminating O_2_ or reducing its local concentration, removing catalytic metal ions, and eliminating key reactive oxygen species (ROS) like O^2-^ and H_2_O_2_. They also scavenge initiating radicals such as OH^−^, RO^−^, and RO^2-^, interrupt the chain reaction of initiated sequences, quench or scavenge singlet oxygen, and boost endogenous antioxidant defenses by upregulating genes that encode antioxidant enzymes. Additionally, antioxidants repair oxidative damage caused by radicals, enhance the elimination of damaged molecules, and avoid repairing excessively damaged molecules to minimize the risk of mutations (Tchimene et al., 2016a). Lupeol contains a single hydroxyl group and a large, apolar skeleton, which allows it to interact with and stabilize free radicals. This amphiphilic nature enables it to penetrate cell membranes and exert antioxidant effects (Park et al., 2023).

The study on DPPH (1, 1-diphenyl-2-picrylhydrazyl) free radical scavenging revealed that lupeol exhibited higher antioxidant activity at elevated concentrations compared to ascorbic acid, with percentages of 88.40% and 82.37% at 800 μg/mL, respectively. Similarly, in the FRAP (Ferric Reducing Antioxidant Power) assay, lupeol demonstrated a higher value of 2.314 ± 0.06 at high concentrations, surpassing that of ascorbic acid even at 1,000 μg/mL. Lupeol effectively scavenged hydrogen by donating electrons to hydrogen peroxide. The findings indicate that lupeol has strong ABTS (2,2′-Azinobis-(3-Ethylbenzthiazoline-6-Sulfonic Acid)) scavenging activity and inhibits lipid peroxidation in the human body. Furthermore, pretreatment with lupeol increased the activity of CAT (catalase), suggesting this might be the mechanism behind the observed reduction in lipid peroxidation (Tchimene et al., 2016b).

Lupeol has demonstrated beneficial antioxidant properties in a streptozotocin (STZ)-induced hyperglycemic rat model, notably increasing the production of Superoxide Dismutase 2 (SOD-2) and Heme Oxygenase-1 (HO-1). Its antioxidant effects were studied in male Sprague-Dawley rats treated with streptozotocin (STZ) and aluminum chloride (AlCl3), revealing elevated antioxidant levels in the cerebellar cortex, including catalase, superoxide dismutase, thiobarbituric acid reactive substances (TBARS), and glutathione (Beserra et al., 2019). Further research confirmed the antioxidant capabilities of lupeol and its derivatives (isolated from the stem bark of *Crataeva nurvala*) in triton-induced hyperlipidemic adult male rats of the Charles Forest strain, showing a reduction in superoxide anions and hydroxyl free radicals following the administration of lupeol and its chalcone derivatives (Srivastava et al., 2013). Additionally, Santiago et al. verified lupeol’s antioxidant properties (extracted from Ficus pseudopalma Blanco) in scavenging nitric oxide (NO), hydroxyl, and superoxide radicals (Santiago and Mayor, 2014). In diabetic rats, lupeol was also shown to influence hepatic glucose metabolism, resulting in improved liver glucose levels and enhanced antioxidant functions (Ramu et al., 2016). Sunitha et al. studied the antioxidant capacity of lupeol and its chalcone lupeol linoleate through oral administration in a rat model of hepatotoxicity, observing significant improvements in liver antioxidant levels (Sunitha et al., 2001). Furthermore, lupeol, its ester, and lupeol linoleate were found to inhibit fetal cardiotoxicity induced by oxidative stress in cyclophosphamide-treated rats, showcasing lupeol’s antioxidant potency (Sudharsan et al., 2005).

Lupeol, extracted from various sources like *Crataeva nurvala* and *Ficus pseudopalma* Blanco, demonstrates antioxidant effects by increasing enzymes like Superoxide Dismutase 2 (SOD-2), Heme Oxygenase-1 (HO-1), Catalase, and reducing substances like thiobarbituric acid reactive substances (TBARS) and glutathione. These effects were observed in streptozotocin (STZ)-induced hyperglycemic rats, triton-induced hyperlipidemic rats, and rats with hepatotoxicity induced by cyclophosphamide. Overall, lupeol and its derivatives show promise in enhancing antioxidant defenses in various biological contexts.

### 5.2 Anticancer and apoptotic efficacy

Apoptosis, also known as programmed cell death, is a mechanism that has been conserved throughout development and is crucial to the growth and homeostasis of tissues in organisms. But in diseased states, like cancer, cells lose their capacity to perform apoptosis-induced death, which results in uncontrolled proliferation. It is frequently discovered that cancer cells overexpress a large number of proteins that are crucial for preventing the apoptotic cascade from being activated. Cells can evade programmed cell death in a number of ways, one of which is the overexpression of anti-apoptotic molecules. Small molecule inhibitors (SMI) that disrupt the anti-apoptotic pathways of proteins like B-cell lymphoma 2 (Bcl-2), B-cell lymphoma extra large (Bcl-xL), induced myeloid leukemia cell differentiation protein (Mcl-1), Bcl-2-like-protein-2 (BCL2L2/Bcl-w), and Bcl-2-related protein A1 (A1/Bfl1) were developed at a fast pace in the drug discovery field (Mohammad et al., 2015).

Apoptosis and anti-apoptosis are the two categories into which genes can be divided. The p53 gene, a type of tumor suppressor gene that can modify critical cellular processes, including apoptosis, by encoding 53 KDa nuclear phosphoprotein, is found on the short arm of the human chromosome. Proper upregulation of the gene mentioned above can increase the rate of apoptosis in breast cancer cells. Bcl-2 is another apoptotic gene that is present in both the inner and outer membranes of mitochondria. Cell apoptosis can result from caspase gene activation caused by low expression of the Bcl-2 gene. Research shows that the balance between anti-apoptotic and pro-apoptotic proteins, such as BCL2 and BAX, works well for the proliferation of cancer cells (Fatemizadeh et al., 2022; Yoon and Yoon, 2012). There is a clear link between the cell cycle and cancer because the machinery of the cell cycle regulates cell proliferation. This is in sharp contrast to normal cells, which divide only a limited number of times before entering growth arrest, while cancer cells continuously divide (Baychelier and Vieillard, 2013).

**FIGURE 3:**
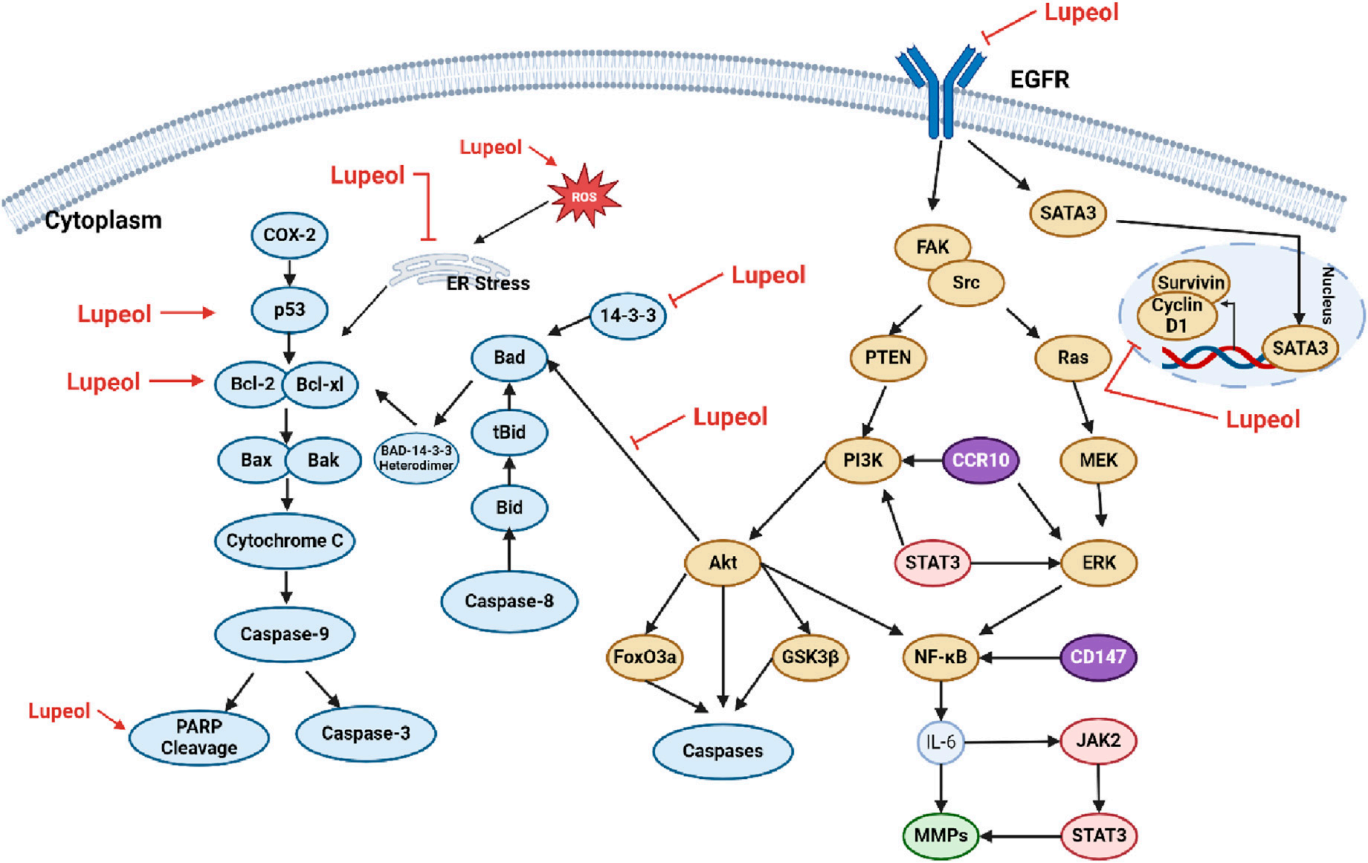
Mechanism of lupeol on the apoptotic pathway.

Numerous investigations have shown that lupeol can trigger apoptosis in cancer cells through various mechanisms. Cancer cells often evade apoptotic programs due to an imbalance between pro-apoptotic and anti-apoptotic proteins. For instance, in different types of cancer cells, such as SMMC7721, HepG2, LNCaP, A431, HEp-2, UPCI: SCC-131, SW480, HCT116, MCF-7, and HeLa cells, lupeol has been shown to activate the mitochondrial-mediated apoptotic pathway (Liu et al., 2021a). Specifically, lupeol therapy may increase reactive oxygen species (ROS) levels, enhance the Bax: Bcl-2 ratio, and subsequently promote PARP (poly (ADP-ribose) polymerase) cleavage. This process can activate caspases and initiate the apoptotic execution phase (Prasad et al., 2007; Bhattacharyya et al., 2017). Numerous studies have demonstrated the significant potential of lupeol in the prevention and treatment of various malignancies, including osteosarcoma, colorectal cancer, bladder cancer, lung cancer, and liver cancer. The molecular pathways involved include the induction of apoptosis, suppression of cell proliferation, and inhibition of cancer cell migration and invasion (Min et al., 2019; He et al., 2018; Tarapore et al., 2013). The mechanism of lupeol on the apoptotic pathway can be seen on Figure 3.

Molecularly targeted medicines have changed the treatment of cancer by allowing for the individualized management of cancers whose growth is driven by particular mutations. These targeted medicines are different from typical chemotherapies in that they are made to specifically disrupt the actions of specific signaling proteins whose activity is primarily limited to malignant tissue. Typical chemotherapies are hazardous to both healthy and tumor cells. Molecular targeted therapy, which employs therapeutic monoclonal antibodies or small-molecule medicines as signal transduction inhibitors, has become the basis of precision medicine in the treatment of cancer. These methods are currently used in clinical practice as first-line treatment (Min and Lee, 2022).

Similarly, doxorubicin and lupeol together prevented the growth of tumors. There were no symptoms of toxicity (infection, diarrhea, or weight loss) when lupeol was taken either alone or in conjunction with a low dosage of cisplatin and doxorubicin. The hematoxylin and eosin slice of the histology of the normal organs, including the tongue, heart, liver, spleen, lung, and kidney, revealed no necrosis or appreciable cell death. Furthermore, lupeol exert showed a synergistic impact in conjunction with low-dose chemotherapy medicines, leading to a significant tumor shrinkage. When compared to the use of the chemotherapeutic medications alone, the combination of lupeol and cisplatin/doxorubicin treatment dramatically induced tumor cell death (Lee et al., 2011). The TNBC cell line MDA-MB-231 was used to examine the synergistic effect of lupeol and the 5FU combinatorial method, which demonstrated efficacy in inhibiting proliferation and wound healing capability. Furthermore, MDA-MB-231 cells have a mesenchymal phenotype and are well-known for their multidrug resistance (Mitra et al., 2023).

### 5.3 Cardioprotective and anti-atherosclerotic activities

Endothelial cells, found in the intima of blood vessels, regulate vascular function by responding to hormones, neurotransmitters, and vasoactive substances (Galley and Webster, 2004). If these cells are damaged, it can cause endothelial dysfunction, an early sign of atherosclerosis (Lerman and Zeiher, 2005). Oxidative stress and inflammation are also an important cause of this dysfunction and can lead to cardiovascular disorders such as atherosclerosis (Sandoo et al., 2010; Higashi and Yoshizumi, 2004; Schiffrin and Deng, 1995). Atherosclerotic plaques are heavily dependent on the balance between the recruitment and activation of monocyte-derived macrophages and their clearance from the vessel wall. Macrophages and foam cells in the vessel wall contribute to plaque destabilization and rupture by secreting proinflammatory cytokines and matrix metalloproteinases. The M1 phenotype produces proinflammatory cytokines and reactive oxygen species, which promote atheroma production; in contrast, the M2 condition releases immunosuppressive cytokines and growth factors, which resolve atheroma by angiogenesis and phagocytosis. The imbalance could be responsible for cholesterol crystal production or elimination (Murray et al., 2014; Randolph, 2014).

The poor control of LDL cholesterol is an essential event in dyslipidemia-induced endothelial dysfunction required for cardiovascular disorders such as myocardial infarction and stroke (Higashi et al., 2009). Elevated LDL cholesterol, particularly oxidative LDL, is pro-atherogenic and impairs endothelial function due to its accumulation in the vessel wall, inflammation, endoluminal remodeling, and lipophilic core extrusion to form early atherosclerotic lesions (Ross, 1999). Increased oxidative LDL also increases proteins that facilitate immune cell binding with LDL (Kita et al., 2001) and decreases the eNOS/NO pathway, worsening inflammation and tissue healing (Higashi, 2023). Nonoxidative LDL also plays a role, although not as effectively as oxidative LDL. On the other hand, AD is anti-atherogenic since it stimulates eNOS, transports cholesterol from macrophages to the liver, and decreases oxidized LDL (Cockerill et al., 1995; Yuhanna et al., 2001).

Myocardial infarction (MI) development involves plaque rupture, thrombosis, cardiomyocyte loss, and inflammation, indicating a major global public health issue (Leancă et al., 2022). Thrombosis in an artery or bypass graft can cause cardiac arrest and death (Roth et al., 2020; Golforoush et al., 2020). Current therapies, including thrombolysis, percutaneous coronary intervention, and coronary artery bypass grafting, aim to improve blood flow but can cause complications like bleeding and ischemia-reperfusion damage (Mackman et al., 2020; Doenst et al., 2019). New therapies are needed to preserve heart muscle and prevent heart failure (Sabatine and Braunwald, 2021). Post-MI, the myocardium undergoes inflammation, scar tissue formation, and angiogenesis. Damaged tissue triggers inflammation, forming granulation tissue and attracting immunocytes that release cytokines and chemokines (Fraccarollo et al., 2012; Viola et al., 2021). Myeloid cells spread pro-inflammatory signals like TGF-β/SMAD and Wnt/β-catenin (Fan and Kassiri, 2021). Fibroblasts produce collagen, and endothelial cells secrete angiogenic factors via PI3K/Akt and JAK/STAT signaling (Fraccarollo et al., 2012; Viola et al., 2021; Jung et al., 2018). Key pathways regulating cardiac repair and hypertrophy include Notch, Nrf2/HO-1, RhoA/ROCK, and Sonic hedgehog (Jung et al., 2018; Contessotto and Pandit, 2021). Promising therapies, like pharmacotherapy, gene therapy, and cell therapy, aim to improve post-MI outcomes by modulating these pathways (Wang et al., 2021; Beliën et al., 2022). Targeting anti-inflammatory and antifibrotic effects and promoting angiogenesis remains crucial for advancing MI treatment (Goumand and Ten Dijke, 2018; Xu G. R. et al., 2020a).

**FIGURE 4:**
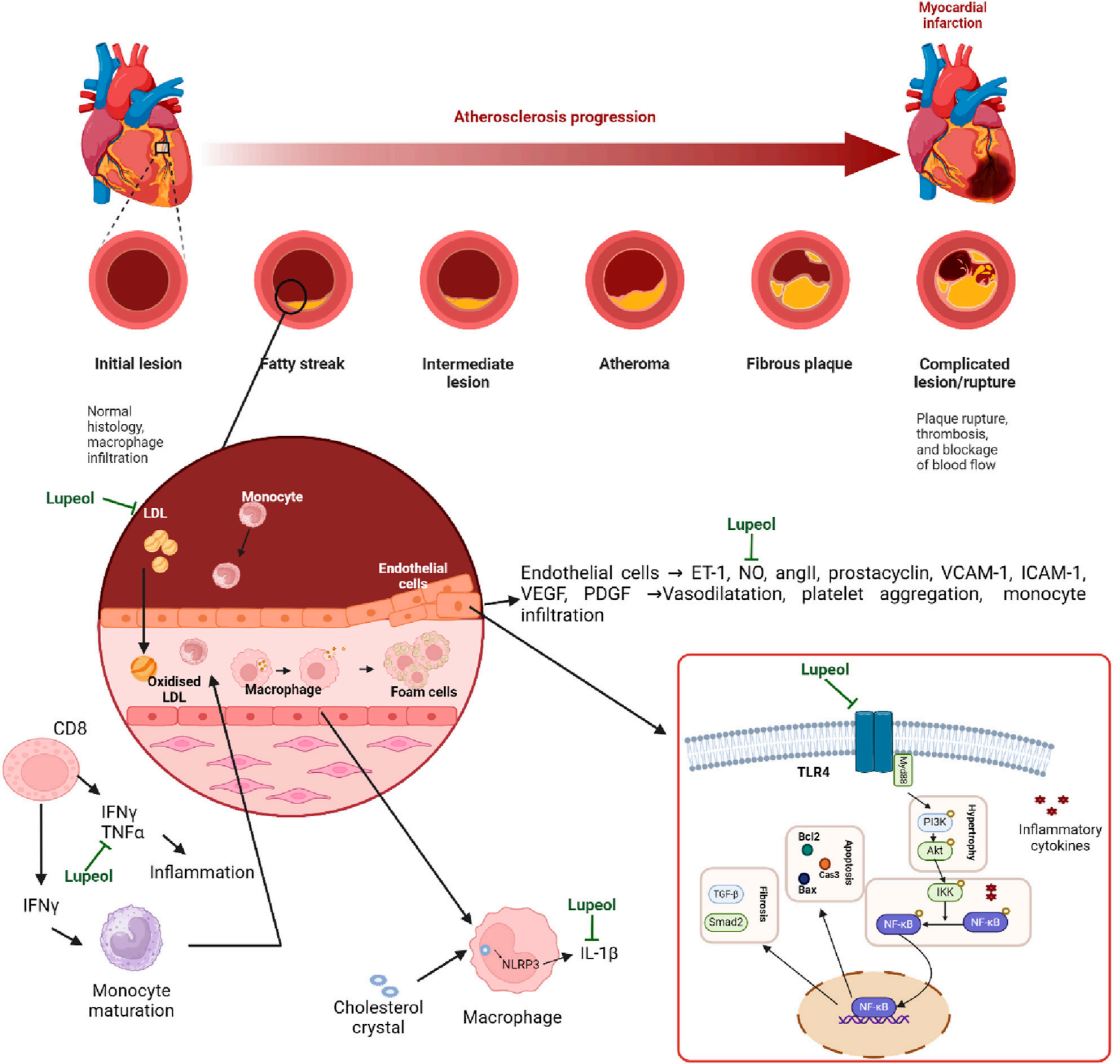
Mechanism of Lupeol’s cardioprotective and anti-atherosclerotic activities.

Lupeol, a natural triterpenoid, has anti-inflammatory and anti-apoptotic characteristics, which may prevent cardiovascular disease and preserve endothelial function due to its antioxidant capabilities (Park et al., 2023; Li et al., 2022). The triterpene structure in lupeol showed changes in hypercholesterolemic rats (decreased activity in Na(+), K(+)-ATPase, Ca(2+)-ATPase, and Mg(2+)-ATPase). Triterpene therapy corrected these levels, preventing hypertrophic cardiac histology and restoring normal ultrastructural architecture (Sudhahar et al., 2007). The hydroxyl group at C3 on lupeol structure plays a critical role in the antioxidant activity of lupeol. It helps in scavenging free radicals and reducing oxidative stress, which is a major factor in cardioprotective effects. This action protects the heart tissue from damage caused by reactive oxygen species (ROS) (Chaudhary et al., 2023). For example, Perreira et al. found that in keratinocytes, lupeol treatment resulted in the activation of Akt, p38, and Tie-2, which are signaling proteins involved in cell proliferation and migration, angiogenesis, and tissue repair (Perreira Bessera et al., 2018). Sudharsan et al. also found that lupeol decreases oxidative stress and exerts its protective effects against CP-induced cardiotoxicity through the direct scavenging of free radicals, which may regulate mitochondrial function (Sudharsan et al., 2006). Xu G. R. et al. (2020a) found that lupeol inserts its cardioprotective effects by inhibiting the activation of the MyD88-dependent pathway, as shown by the reduced protein expression of TLR4, MyD88, and p-NF-κB P65. In addition, gene silencing of TLR4 reduced the levels of pro-inflammatory cytokines, including IL-1β, COX2, and TNF-α (Xu G. R. et al., 2020a). More evidence of lupeol’s cardioprotective and anti-atherosclerotic activities can be seen in Tables 4, 5. These findings show that lupeol exhibits significant potential as a cardioprotective agent due to its anti-inflammatory, anti-apoptotic, and antioxidant properties. By preserving endothelial function and modulating cholesterol dynamics, lupeol may play a crucial role in preventing and treating cardiovascular diseases. Future research should focus on clinical trials to establish optimal dosing, efficacy, and safety in diverse populations, paving the way for novel therapeutic applications in cardiovascular health. The mechanism of Lupeol’s cardioprotective and anti-atherosclerotic activities can be seen on Figure 4.

**TABLE 4:** *In Vitro* studies on the pharmacological properties of lupeol.

Pharmacological effects	Concentration of lupeol	Subject	Result	References
Anti-atherosclerotic	10–50 μM**	PBMC	↑ IL-10, ↑ TGF-β, ↓ IL-12, IL-6; ↓M1 activation marker HLA-DR, ↓NF-κB, ↑FGF-2, TGF-β1, collagen III levels	Saha et al. (2020)
Anticancer	0, 6, 12 and 24 µM**	A427 lung cancer cells and MRC-5	↑ Bax; ↓ Bcl-2; ROS in the A427 cancer cells ↑; and ↓MMP	He et al. (2018)
	0, 5, 10, and 15 μM**	U-2 OS cells	↓p-p38; ↓β-catenin; ↓ Ras, p-Raf-1 and p-p38 expression in U-2 OS cell	Hsu et al. (2019)
	10–100 µM**	H1299, A549, H460, H292 human NSCLC	AKT, as well as the MAPK proteins by EGFR ↑	Min et al. (2019)
	*50* * * µM**	(UPCI:SCC131 and UPCI:SCC084)	↓phosphorylation of EGRF; ↓AKT; ↓IκB NF-κB; and ↓COX-2	Rauth et al. (2016)
	0; 5; 10; 15; 30; 60; 90; 120; 150; 200 µM**	MHCC-LM3	↓CD13+HCC; ↓Hepatosphere Formation	Lee et al. (2011)
	0–50 μM**	HEp-2, and UPCI:SCC-131 cells	↑CDKN2A; ↓CyclinD1; ↓Ki67; ↑p53; ↑Bax; and ↑Caspase-3c	Bhattacharyya et al. (2017)
	0–20 µM**	MHCC-LM3	↑c-MET and EphA2; ↑Phosphorylation	Mitra et al. (2023)
	20–60 μM**	Mel 928, Mel 1,241 and Mel 1,011	Cyclin D1 and c-myc↓ causes apoptosis, nuclear antigen and Ki-67↑ and invasion marker osteopontin	Tarapore et al. (2010)
	20–40 μM**	LNCaP and DU145	↓CaP cells; ↓RNA transfection and GSK3b activity; ↓MMP-2	Saleem et al. (2009)
	20–40 μM**	DLD 1 and HCT 116	↓Wnt/β-catenin	Tarapore et al. (2013)
	0–200 μM**	HCCLM3 cells, HepG2 and Huh-7	↓BDNF ser-9-; ↓GSK-3β, ↓Akt1; ↓PI3K β-catenin; ↓c-Myc and Cyclin D1 mRNA; ↓p-Akt and PI3K (p110α); ↓GSK-3β function in HepG2 cells	Zhang et al. (2015)
Anti-cholesterol	1–10 mM**	HepG2-Lipo cells	↓ TG, cholesterol biosynthesis, lipoprotein secretion	Itoh et al. (2009)
Antidiabetic	10, 20, 50, 100 μg/mL**	3T3-L1 Preadipocyte Cells	↓Triglyceride accumulation, induced lipid accumulation	Uddin Ahmed et al. (2012)
	15–240 μg/mL**	Recombinant human DPP-IV	↓DPP-IV activity and glycation process, ↑glucose uptake	Matsabisa et al. (2020)
	0.02–200 μg/mL**	C2C12 Myocytes Cells	↓Blood glucose, ↓α-glucosidase	Deutschländer et al. (2011)
	0/25–1 g/L**	Mice Abdominal Muscle	↑Glucose uptake, ↑glucose oxidation, ↑glycogenesis, insulin secretion	Gray et al. (2000)
	*N/A	NPCs	↓High-glucose-induced apoptosis	Guo et al. (2018)
	10 μm**	L6 skeletal muscle cells	↓Blood glucose	Khan et al. (2014a)
Anti-inflammatory and Anti-pyroptotic	5, 10, 20, 30 μM**	Mice BMDMs	↓ M1 polarization, ↓cell viability, ↓ NLRP3 and pro-IL-1β, ASC oligomerization, cleaved Caspase-1, mature IL-1β, and GSDMD-NT, ↓NF-κB pathway	Xiong et al. (2024)
Cardiac Hypertrophy protection	50 μg/mL**	NRCMs	protective effect on cell viability, ↓PI3K and Akt	Li et al. (2022)
Dermatoprotective	0.1, 1, 10, and 20 μg/mL**	human keratinocytes cells	↑ MMP-2 and↓NF-κB expression in keratinocytes ↑ keratin 16 expression ↑ Akt, p38, and Tie-2	Beserra et al. (2018)
	0.4–50 μM**	NHDF-neo cells	↓MMP-1, -2, -3, ↓p-p53, ↓p21, ↓p16, and ↓SA-β-gal	Park and Park (2019)
Hepatoprotective	10 μM**	Hep cells	↑ Bcl-2, SOD, GSH and NADPH	Kumari and Kakkar (2012)
Renoprotective	125 μM**	LLC-PK1 cell	↓ MAPK-caspase-3	Lee et al. (2015)
Wound healing, Cardiac Hypertrophy protection	0.1 and 1 μg/mL**	human neonatal foreskin keratinocytes and fibroblasts	↑Akt, p38, and Tie-2	Pereira and Beserra (2020)

Abbreviation: BMDM (Bone-marrow-derived macrophage); DPP IV (Dipeptidyl peptidase-IV); IL (Interleukin); NHDF-neo (Neonatal normal human dermal fibroblast cells); NPCs (Rabbit Nucleus Pulposus Cells); NRMC (neonatal rat cardiomyocyte); PBMCs (Peripheral blood mononuclear cells); Hep (heptocytes); SOD (Superoxide dismutases); GSH (Glutathione); NADPH (Nicotinamide adenine dinucleotide phosphate); MAPK (Mitogen-activated protein kinases); MMP-2 (Matrix Metalloproteinase-2); NF-κB (Nuclear Factor kappa-light-chain-enhancer of activated B cells); p38 (p38 Mitogen-Activated Protein Kinase); Tie-2 (Tyrosine kinase with immunoglobulin-like and EGF-like domains 2); SA-β-gal (Senescence-Associated Beta-Galactosidase); Bcl-2 (B-cell lymphoma-2); Wnt (Wingless); BDNF (Brain-Derived Neurotrophic Factor); GSK-3β (Glycogen Synthase Kinase 3 Beta); PI3K (phosphatidyl inositol 3-kinase); EGFR (epidermal growth factor receptor); TG (Triglycerides); Akt/PKB (protein kinase B); NLRP3 (NOD-like receptor protein 3).

**the dose indicated by original article.

*N/A: information is not provide by the original article.

**TABLE 5:** *In Vivo* studies on the pharmacological properties of lupeol.

Pharmacological effect	Cell lines/Animal models	Inducer	Lupeol dose	Treatment duration	Mechanism of action	References
Analgesic	Male Swiss Webster mice	carrageenan (100 μg per paw; 20 μL)**	50 mg/kg and 100 mg/kg**	7 days	↓TNF-a and IL-1b	De Lima et al. (2013)
	Male Wistar albino rat	1% acetic acid (1 mL/100 g)	100 mg/kg	7 Days	↓PGE2, inflammatorycytokines	Rathinavel et al. (2021)
Anticancer	Male Athymic (nu/nu) nude mice	Implanted Melanoma cells	40 mg/kg body wt**	20 days	↓Cyclin D1 and c-myc; causes apoptosis, nuclear antigen; ↑Ki-67 and invasion marker osteopontin	Tarapore et al. (2010)
	Male Golden Syrian hamsters	0.5% DMBA (0.5%, third weekly)	50 mg/kg (po) 1 week before DMBA treatment**	7 days	↓p53; ↓Bcl-2; ↑Bax; ↑caspase 3 and 9	Manoharan et al. (2012)
	Nude mice***	2 × 10^6 SMMC7721 cells suspended in 0.2 mL PBS	20 mg/kg or 80 mg/kg**	30 days	↓ Cell viability; ↑ caspase-3 activity; ↓ tumorigenicity via TRAIL pathway	He et al. (2018)
	Nude mice***	1 × 10^6 MHCC-LM3 cells	1 mg/animal**	30 days	↓Self-renewal ability; ↓tumorigenicity; ↑ HCC cells	Lee et al. (2011)
	Swiss Albino mice***	DMBA (50 mg/kg, po)	25 mg/kg (po) for 1 week after DMBA**	7 days	Restore antioxidant and mitochondria function; ↓Bcl-2; ↑Bax; ↑Caspase 3	Prasad et al. (2007)
Antidiabetic	Albino Rats***	Alloxan 150 mg/kg**	200 mg/kg**	*N/A	↓ blood glucose, ↓OS	Malik et al. (2019)
	Female and Male Wistar Rats	Alloxan 120 mg/kg**	100 mg/kg**	28 days	β-cell regeneration, ↑hepatic glucose utilization	Ramu et al. (2016)
	Male Albino SD Rats	Alloxan 160 mg/kg**	250, 500 mg/kg**	*N/A	↓blood glucose	Uddin Ahmed et al. (2012)
	Male Albino Wistar Rats	Sucrose****	25 mg/kg	30 days	↑ IRS-1 mRNA level, ↑ Akt protein level	Gayathri et al. (2019a)
	Male Albino Wistar Rats	High-fat diet (cholesterol 3%, cholic acid 1%, coconut oil 30%, standard rat feed 66%, 30% sucrose)**	25 mg/kg**	30 days	↓high-fat diet and SREBP-1c levels	Gayathri et al. (2019b)
	Male Albino Wistar Rats	Sucrose****	25, 50 mg/kg**	30 days	Regulation of insulin signaling molecules (IR and GLUT 4)	Gayathri et al. (2019c)
	Male Albino Wistar Rats	STZ 55 mg/kg**	50, 100 mg/kg**	21 days	↓carbohydrate hydrolytic enzymes, ↑glycogen regulatory enzymes, ↑lipid profile	Soni et al., 2018
	Male CD-1 Mice	Glucose 2 g/kg**	10 mg/kg**	*N/A	↓lipid accumulation, ↓blood glucose	Giacoman-Martínez et al. (2019)
	Male ICR Mice	STZ 60 mg/kg**	10 mg/kg**	*N/A	↓carbohydrate-digesting enzymes	Lee et al. (2021)
	Male SD Rats	STZ 30 mg/kg	10 mg/kg**	21 days	↓ plasma glucose level, ↑ antioxidant enzymes	Hashmi et al. (2018)
	Male Swiss Albino Mice	STZ 150 mg/kg**	25, 50, 75, 100 mg/kg**	7 weeks	↓ hyperglycaemic, ↓ OS, reversed TNF-α, IL-1β, ↓ NF-κB	Das et al. (2022)
	Male Swiss Mice	Cornstarch 2 g/kg**	300 mg/kg**	*N/A	↓α-glucosidase, ↑glucose uptake	Pereira et al. (2015)
	Male Wistar Rats	STZ****	200, 400, 800, 1,600, 3,200 mg/kg	14 days	↓ glucosidase enzyme, reverse the fasting hyperglycaemia	Liyanaarachchie et al. (2021)
	Rats***	STZ****	100 mg/kg**	*N/A	↓Blood glucose	Reddy et al. (2009)
	Wistar Rats***	STZ****	20, 30, 40 mg/kg**	21 days	Pancreatic regeneration, ↑proteins synthesis, ↑serum insulin	Gupta et al. (2012)
Anti-inflammatory and Anti-pyroptotic effects	Male Balb/c mice	250 μg αMHC peptide + CFA**	25–50 mg/kg/day**	21–60 days	↓ M1 polarization, ↓ PPARα/LACC1/NF-κB pathway	Xiong et al. (2024)
Antiproliferative and anti-inflammatory	Male Albino Wistar rats	BBN (150 mg, twice weekly) for 8 weeks and then DMA (100 ppm) for 28 weeks	50 mg/kg (po) post to BBN treatment**	28 weeks	↓ tumor growth; ↑ PTEN; ↓COX-2; ↓NF-kB; ↓TNF-a	Prabhu et al. (2016)
Antiurolithic	Male Wistar rats	0.75% EG in drinking water**	50 mg/kg**	28 days	↑ antioxidant, ↓ oxalate	Sudhahar et al. (2008a)
Antiurolithic and nephroprotective	Male Albino Wistar Rats	0.75% EG in drinking water**	50 and 100 mg/kg**	28 days	↓ SCr, UA, Ca, PO₄³⁻, and CaC_2_O_4_	Manjula et al. (2012)
Cardiac Hypertrophy protection	Rats***	pressure overload ****	50 mg/kg**	4 weeks	↓ cardiac fibrosis, ↓ TLR4-PI3K-Akt-NF-κB	Li et al. (2022)
Cardioprotective	Male albino rats of Wistar	CP 200 mg/kg bw**	50 mg/kg b.w.**	10 days	↓ oxidative stress, ↑Cardioprotective	Sudharsan et al. (2006)
	Male BALB/C mice	CVB3 10^2 TCID50, i.p**	50–100 mg/kg**	21 days	↓TLR4, MyD88 and p-NF-κB P65. ↓ pro-inflammatory cytokines, ↓IL-1β, ↓COX2, ↓TNF-α	Xu et al. (2020b)
Dermatoprotective	Male Wistar rats	STZ 50 mg/kg**, i.p	0.2% w/w lupeol cream**	14 days	↓ NF-κB, ↑ FGF-2, TGF-β1, and collagen III, ↓ IL-6, ↑ IL-10, ↑ mRNA expression levels of HIF-1α, SOD-2, and HO-1	Beserra et al. (2019)
	Male Wistar rats	Normoglycemic (without inducer)	0.2% and 0,4% w/w lupeol cream**	14 days	↓ TNF-α, IL-1β, and IL-6, ↓ Gene and protein NF-κB expression, ↑ IL-10, ↑ VEGF, EGF, TGF-β1, ↑ Collagen synthesis	Beserra et al. (2020)
	SKH1 male mice	DMBA(390 nmol/0.1 mL acetone) and UVB radiation 2 times/week for 5 min reaching a total dose of around 200 J/m2**	Lupeol and HPGCD mixture 1:1	6 weeks	↑ TEWL, erythema, skin hydration, and sebum content	Minda et al. (2015)
Hepatoprotective	Male Albino Rats Wistar strain	AFB1 1 mg/kg b.m**	100 mg/kg b.m**	10 days	↑ GSH, ↑ SOD, ↑ Antioxidant, ↓ALT, ↓AST, ↓ LDH, ↓ ALP	Preetha et al., 2006
	Male ICR mice	GaIN/LPS 800 mg/kg**	25, 50, and 100 mg/kg**	N/A*	↓lethal liver damage	Kim et al. (2014)
Nephroprotective	Female Albino Rats	cadmium chloride 1 mg/kg for 15 days**	40 mg/kg**	15 days	↓ peroxidative damage, exerting cytoprotective	Nagaraj et al. (2000)
	Male Albino Rats Wistar Strain	4% cholesterol and 1% cholic acid for 30 days**	50 mg/kg**	the last 15 days along with the inducer	Reactivated the functions of renal marker enzymes	Sudhahar et al. (2008b)
	Male Albino Rats Wistar Strain	HFD (cholesterol 3%, cholic acid 1%, coconut oil 30%, standard rat feed 66%, and 30%) for 60 days through drinking water**	25 mg/kg and 50 mg/kg**	30 days	Protect the kidney from exogenous chemicals	Kaviya et al. (2019)
Renoprotective	Albino Wistar Rats of either sex	STZ 50 mg/kg**	35 mg/kg and 75 mg/kg**	28 days	↑ GSH, ↑ SOD, ↑ Antioxidant, ↑ Free radical scavenger, ↓ plasma, ↓ urine creatinine, ↓ blood urea nitrogen, ↓ urine protein	Tiwari et al., 2019

Abbreviation: αMHC (alpha-myosin heavy chain); AFB1 (aflatoxin B1); b.m (body mass); CVB3 (coxsackie virus B3); CP (cyclophosphamide); DMBA (7,12-dimethylbenz(a)anthracene); GLUT4 (Glucose Transporter Type 4); HPGCD (hydroxy-propil-gamma cyclodextrin); ALT (Alanine aminotransferase); LDH (Lactate dehydrogenase); IR (Insulin Resistance); OS (Oxidative Stress); SREBP-1c (Sterol Regulatory Element Binding Protein-1c); STZ (Streptozotocin); TCID50 (50% tissue culture infectious dose); TEWL (transepidermal waterloss); EG (ethylene glycol); SCr (serum creatinine); CaC_2_O_4_ (calcium oxalate); UA (Uric acid); NF-κB (Nuclear Factor kappa-light-chain-enhancer of activated B cells); FGF-2 (Fibroblast Growth Factor 2); TGF-β1 (Transforming Growth Factor Beta 1); IL-6 (Interleukin 6); IL-10 (Interleukin 10); HIF-1α (Hypoxia-Inducible Factor 1-alpha); SOD-2 (Superoxide Dismutase 2); HO-1 (Heme Oxygenase 1); VEGF (Vascular Endothelial Growth Factor); EGF (Epidermal Growth Factor); PTEN (Phosphatase and tensin homolog); Bcl-2 (B-cell lymphoma 2); HCC (hepatocellular carcinoma).

*N/A: information is not provide by the original article.

**the dose indicated by original article.

***information about the gender of the sample is not provide by the original article.

****the inducer dose is not provide by the original article.

### 5.4 Antidiabetic actions and glycemic control

Diabetes mellitus (DM) is a metabolic condition that is typically characterized by inadequate production of insulin or dysfunction in the insulin receptors, leading to persistently high levels of glucose in the blood (Gayathri et al., 2019b). The presence of antidiabetic medications for the treatment of diabetes mellitus has specific limitations and is not affordable for developing countries. There is a demand for an antidiabetic medication therapy that can be used in a broader range of cases, is safer, and has a higher level of effectiveness (Lakshmi et al., 2015). Diabetes patients frequently experience vascular problems as a result of multiorgan dysfunction, which affects the abnormal structure and function of the vascular complications (Wei et al., 2022). Lupeol exhibits a wide range of pharmacological effects in both *in vitro* and *in vivo*, including the ability to decrease the risk of diabetes development (Gayathri et al., 2019c).

The evidence regarding the impact of lupeol on the survival of β-cells can be categorized into two broad groups: the trials conducted on isolated islets or insulin-secreting cell lines *in vitro*; and the studies carried out on live animal models of diabetes. Cohort studies have shown that preserving residual β-cell function can guard against the development of diabetic problems. This highlights the importance of treatments that can help maintain β-cell mass and function over a long period of time (Meier, 2008). Lupeol has been identified as an antidiabetic agent in rodent models, which through pancreas regeneration is able to reduce hyperglycemia as demonstrated through laboratory studies. Additional reports on STZ-induced histological studies showed that lupeol treatment for 21 days can rejuvenate the destroyed and severely damaged cells in the pancreas many times over. This reinforces the role of lupeol in regenerating insulin-producing cells and its insulinotropic role (Gupta et al., 2012). Reduction of lipid peroxidation and protein carbonylation is one of the molecular mechanisms by which lupeol preserves and regenerates β-cells as well as increases insulin levels. Elevated levels of peroxidation of lipids in rats led to the formation of MDA in the biological system. Increased levels of NO result in an enhanced concentration of peroxynitrite in the body, consequently leading to elevated amounts of RNS. This has been confirmed by various studies describing the relationship between decreased oxidative stress activity and antioxidant pancreatic enzymes (e.g., CAT, GPx, and SOD) in diabetic conditions (Ghosh et al., 2018; Tsai et al., 2016). In normal physiological function, these antioxidants play an important role in eliminating ROS. GPx reduces H_2_O_2_ to H_2_O and CAT catalyzes the decomposition of H_2_O_2_ to H_2_O and O_2_. The low availability of NADPH in diabetes, which leads to decreased CAT activity, can be improved after treatment with lupeol, as well as improved SOD, GSH, and GPx in diabetic rats (Malik et al., 2019). Subsequently, the administration of lupeol substantially improved the activity of glutathione-S-transferase while reducing the levels of GSH and thiobarbituric acid-ROS. These findings indicate that lupeol has the potential to drive pancreatic regeneration by enhancing protein synthesis (Alqahtani et al., 2013). Xanthine oxidase is an enzyme that catalyzes the conversion of xanthine to uric acid, hence generating superoxide radicals. Current investigations have found that treatment with lupeol can lower the level of xanthine oxidase that is elevated in the hyperglycemic conditions (Das et al., 2022).

Hydrogen peroxide (H_2_O_2_), superoxide anion radicals (O_2_), and hydroxyl radicals (OH) are significant reactive oxygen species (ROS) produced when oxidative stress levels rise. These species have a substantial impact on the destruction of pancreatic β-cells (Malik et al., 2019). In diabetes, the excessive production of RNS/ROS caused by the oxidation of glucose and free fatty acids (FFA) within cells leads to the regulated cell death of β-cells through the intrinsic pathway (Ghorbani et al., 2019). β-cells are particularly susceptible to oxidative stress due to their intrinsically limited antioxidant capability (Lenzen, 2008). Furthermore, prolonged exposure of β-cells to high levels of FFA results in endoplasmic reticulum (ER) stress. This stress leads to programmed cell death or apoptosis by increasing the production of CCAAT-enhancer-binding protein homologous protein (CHOP) and activating caspase-12 and c-Jun N-terminal kinase (JNK), which is a type of mitogen-activated protein kinase (MAPK) (Lytrivi et al., 2018).

Protein tyrosine phosphatase 1B (PTP1B) significantly contributes to the suppression of insulin function, the progression of type 2 diabetes, and the development of obesity (Na et al., 2009). In a recent study, Na et al. (2009) demonstrated that lupeol, with an IC_50_ value of 5.6 μM, effectively suppresses the activity of PTP1B. This study proposed that lupeol has therapeutic promise in mitigating other disorders associated with insulin resistance. The efficacy of lupeol in preventing the development of diabetes has been studied by multiple researchers using animal models of diabetes (Ortiz-Andrade et al., 2007; Narváez-Mastache et al., 2008). Lupeol has gone through chemical testing and has been found to be a highly effective scavenger of free radicals (Brimson and Tencomnao, 2012; Tchimene et al., 2016b). Diabetes was linked to the upregulation of pro-apoptotic genes (e.g., caspases) and the downregulation of anti-apoptotic genes (e.g., Bcl-2 proteins) in β-cells, which was caused by glucotoxicity, lipotoxicity, and chronic oxidative stress. The diabetes pathophysiology is linked to the increased amounts of inflammatory cytokines, including IL-1β and TNF-α, which are responsible for attracting lymphocytes and macrophages to sites of inflammation. The control of inflammation is linked to NF-κB, a crucial transcription factor. Oxidative stress triggers the activation of NF-κB, which causes the p65 subunit to move from the cytoplasm to the nucleus by releasing it from phosphorylated IκBα. The latest research indicates that lupeol effectively reduces the movement of the p65 subunit into the nucleus in pancreatic tissues, demonstrating its anti-inflammatory characteristics (Rashid et al., 2017). Lupeol effectively inhibited the DNA degradation caused by caspase activation in the pancreas of diabetic mice induced by STZ. This was achieved by lowering the expression of cleaved caspase-8, cleaved caspase-3, TNF-R1, and TNF-α, which are all involved in the apoptotic pathway associated with TNF-α (Sinha et al., 2019). Based on histological assessment, lupeol can effectively restore the adverse effects of STZ-induced pancreatic islet damage from hyperglycemic conditions leading to reduced insulin-secreting pancreatic β-cells (Matveyenko and Butler, 2008). Gandhi et al. (2012) examined experimental findings *in vivo* involving diabetic rodents and revealed a significant upregulation of β-cell granulation and alterations in cellularity. There was an increase in insulin-immunoreactive β-cells and hypoglycemic activity nearly comparable to that observed in the group treated with oral sulfonylureas, as determined by immunohistochemical reactions of pancreatic islets of diabetic rats administered lupeol-containing extracts (200 and 400 mg/kg). These results suggest that the lupeol-containing extracts have an insulin secretory function.

Inhibition of carbohydrates digestive enzymes, particularly α-amylase and α-glucosidases, is commonly used to decrease the rate at which carbohydrates are digested and absorbed. This method is effective in lowering blood glucose levels and mitigating the possibility of developing diabetes and other metabolic syndromes (Barber et al., 2022). Based on enzyme kinetics studies that have been conducted, lupeol could mediate the inhibitory action of α-glucosidase and α-amylase through a non-competitive inhibition pattern that leads to a reduced rate of product formation from the binding of lupeol to free α-glucosidase protein or pNPG a-glucosidase complex (Ibrahim et al., 2016). Insulin receptor substrate (IRS) proteins are cytoplasmic adaptor proteins that regulate signaling complexes downstream of cell surface receptors. IRS-1 and IRS-2 are ubiquitously expressed, mitogenesis-dependent master mediators of glucose and insulin metabolism and, in most cell types, play an important role in the regulation of glucose transport (Landis and Shaw, 2014; Litherland et al., 2001). IRS-2 plays an important role in the insulin signaling cascade (Hegarty et al., 2003). In an *in vivo* study, it was shown that IRS-2 knockout resulted in the development of diabetes due to hepatic IR combined with progressive loss of pancreatic β-cells. There is a significant increase of IRS-2Ser636 as the phosphorylated form of IRS-2 in the liver of diabetic mice. With the administration of lupeol, serine phosphorylation of IRS-2 was reduced, indicating its prevention against oxidative stress induced by high levels of sucrose and fat (Jayaraman et al., 2019; Togashi et al., 2014).

Hyperglycemia in diabetic conditions can be reduced by the action of insulin on glucose uptake in muscle and adipose tissue (Huang et al., 2018). Therefore, translocation of glucose transporter type 4 (GLUT-4) to the plasma membrane of the 3T3-L1 adipocytes, initiated by the combination of insulin and insulin receptor, can increase glucose uptake into cells, which leads to tyrosine phosphorylation of insulin receptor substrate 1 (IRS-1) and then results in phosphoinositide 3-kinase (PI3K) expression. PI3K expression targets the serine/threonine kinase AKT and the atypical protein kinase C isoforms ζ and λ (PKC ζ/λ) that can stimulate GLUT4 translocation to the PM (Ramachandran and Saravanan, 2015). Another major signaling mechanism that regulates glucose uptake is 5′AMP-activated protein kinase (AMPK) that inhibits anabolic processes and promotes catabolic processes with energy release and decreased blood glucose level in the body (Garcia and Shaw, 2017). In a recent study conducted by Lee and Han (2022), the researchers examined the uptake of 2-deoxyglucose to determine the impact of lupeol on glucose uptake in 3T3-L1 adipocytes. It was discovered that lupeol can increase glucose absorption in a manner that depends on its concentration. The researchers also investigated the impact of lupeol on the activation of IRS-1, PI3K, PKC ζ/λ, and AKT in the insulin signaling pathway. The findings indicate that lupeol greatly increased the phosphorylation of IRS-1 and the activation of PI3K in 3T3-L1 adipocytes. The effect of lupeol on the activation of AMPK and the expression of plasma membrane GLUT-4 in 3T3-L1 adipocytes has prompted an investigation into its mechanism for enhancing glucose uptake.

Using a more practical approach, Devi et al. (2019) tested the antihyperglycemic properties of lupeol on a high-fat diet and sucrose-induced type-2 diabetic (T2D) rats to obtain how this compound effects glucose transporter (GLUT)-4 and As160 protein expression in adipose tissue. Activated IkB kinase pathway attenuates GLUT4 promoter expression in cardiomyocytes and human heart muscle biopsies during elevated FFA and lipotoxicity conditions (Gayathri et al., 2019c). They discovered that lupeol can restore the expression of GLUT-4 in both the cytosol and plasma membrane of adipose tissue in diabetic rats. This could be attributed to the upregulation of signaling molecules by lupeol, which in turn regulates the protein levels of GLUT-4 and As160 as insulin signaling molecules. The ability of lupeol to activate PPARδ/γ by its agonist action has led to the examination of its antidiabetic properties through *in silico* molecular docking studies and *in vitro* assays. PPARδ controls the activity of important genes involved in the uptake and breakdown of fatty acids, as well as the production of uncoupling proteins 2 and 3, and carnitine palmitoyl-transferase 1 and 2 (Matsuura et al., 2013; Ding et al., 2014). PPARγ has a role in the process of adipocyte differentiation and is engaged in the metabolism of lipids and glucose by facilitating their absorption. PPARγ controls the activity of genes that play a role in the movement of lipids, including lipoprotein lipase and apolipoprotein 2, as well as GLUT-1 and GLUT-4 (Wang L. et al., 2014a; Berger et al., 2005). This simultaneous activation of PPARδ/γ can be associated with improved insulin sensitivity and reduced blood glucose in two ways: increased glucose uptake through PPARγ activation, and through its activation of PPARδ can increase lipid uptake and oxidation, thus avoiding weight gain. Lupeol demonstrates potent activity, surpassing even L-165041 and pioglitazone, in inducing changes in lipid and glucose metabolism, hence restoring the existing metabolic imbalance in T2D and other pathologies associated with metabolic disorders (Giacoman-Martínez et al., 2019).

Through its absorption into the hepatic portal vein, the liver has first access to most nutrients, thus playing a unique role in postprandial nutrient metabolism. Nutritional, hormonal, and insulin are factors that can transcriptionally activate SREBP-1c, which is involved in lipid synthesis and glucose metabolism through transcriptional activation mediated by phosphorylation and nuclear translocation of SREBP-1c in the liver (Tian et al., 2016). In another study, lupeol treatment effectively reduced the expression of SREBP-1c protein and restored it to the control level through activating the regulation of insulin signaling molecules and glucose oxidation in the liver conducted on type 2 diabetic rats that previously had elevated levels of the protein in the liver due to a high-fat diet and sucrose-induced hyperglycemia (Sohag et al., 2022). In addition, the synthesized lupeol analogues had a stimulating impact on glucose absorption in L6 skeletal muscle cells (Khan et al., 2014a). Furthermore, an HPLC analysis of *Dicoma anomala* MeOH extract revealed the existence of lupeol, indicating that this biologically active triterpenoid may have a role in the promotion of cellular glucose uptake and its utilization (Matsabisa et al., 2020).

A computer analysis indicated lupeol’s allosteric inhibition effect on PTP1B. Lupeol’s hydrophobic characteristics, attributed to its single hydroxyl group, are posited to be pivotal in its interaction with PTP1B’s allosteric hydrophobic sites, functioning as a negative regulator of insulin signaling pathway binding sites and consequently enhancing insulin response (Jin et al., 2016; Thareja et al., 2010). According to the early structure-activity relationship (SAR), lupeol can augment nitric oxide inhibition, with its derivatives exhibiting differing degrees of action. A carbonyl group at C-3 of lupeol enhances this inhibition, but a hydrophobic methyl ester at C-3 diminishes it. Substitutions on the phenyl ring of the indole structure also enhance the action. Alkyl and halogen substituents on the indole ring significantly enhance nitric oxide inhibition, whereas electron-donating groups further amplify this effect. Despite the inactivity of the derivatives against IL-1β, these results indicate that lupeol derivatives may hold potential for the development of effective NO inhibitors. Furthermore, investigating these compounds may uncover possible antidiabetic capabilities, considering the association among inflammatory pathways and diabetes (Bhandari et al., 2014).

### 5.5 Anti-inflammatory and immunomodulatory effects

An immunomodulator is a chemical that modifies, changes, or supports the regulation of an organism’s immune system (Maurya et al., 2012). Through a variety of processes, one of the secondary metabolite groups found in medicinal plants, triterpenes, exhibits immunomodulatory characteristics. As a pentacyclic triterpene, lupeol, which is the form of lupan in which a hydroxyl group has taken the place of the hydrogen atom at position 3b. By promoting T-lymphocyte proliferation and enhancing macrophage phagocytosis, lupeol demonstrated immunomodulatory action (Renda et al., 2022).

Based on previous study, increased concentrations of lupeol would accelerate the proliferation of lymphocytes. The primary factor driving the process of lymphocyte proliferation was an active substance called an antigen that was confined within the membranes of T and B cells. The primary cell involved in reacting to adaptive immunity is the lymphocyte. APC (antigen-presenting cell) bonded onto the T-cell receptor (TCR) of the complex peptide MHC, causing proliferation in lymphocytes. Stimulation signals were then given through the interaction of CD28 with its ligands. An activated T-cell of a lymphocyte would generate cytokines such as interleukin-1 (IL-1), interleukin-2 (IL-2), and interferon-γ (IFN-γ) (Wahdaningsih et al., 2021).

The primary source of inflammatory mediators is macrophages (Li et al., 2020). When macrophages come into contact with infections, they release proinflammatory cytokines as well as reactive oxygen and nitrogen species, which support the immune system’s activation. Lipopolysaccharide (LPS) and interferon-γ (IFN-γ) are examples of inflammatory stimuli that cause an inflammatory phenotype in macrophages that enhances the TH1 effector response. This type of macrophage is known as “classically activated” macrophages. On the other hand, when macrophages are stimulated with cytokines like interleukin-4 (IL-4) or IL-13, they enter an “alternative” activation state that is marked by an increased phagocytosis activity and a decreased capacity to produce proinflammatory cytokines (Ginhoux et al., 2016).

Lupeol significantly reduced the production of proinflammatory cytokines, including IL-6, TNF-α, and monocyte chemoattractant protein-1 (MCP-1), in the liver. Meanwhile, it increased the expression of anti-inflammatory cytokines, including IL-4 and IL-10, in rats with diet-induced metabolic syndrome (MS). Importantly, lupeol remarkably inhibited M1 macrophages polarization (F4/80+ iNOS+), indicating reduction of inducible nitric oxide synthase, IL-1β, IL-6, and TNF-α expression as M1 markers, while elevated M2 macrophages polarization (F4/80+ CD206+), indicating increment of M2 markers, such as arginase-1, IL-10, CD206, and TGF-β. At the same time, the levels of M1 markers, including inducible nitric oxide synthase, IL-1β, IL-6, and TNF-α, were markedly inhibited, while those of M2 markers, such as arginase-1, IL-10, CD206, and TGF-β (Li J. et al., 2021b). Lupeol also prevented the elevation of pro-inflammatory cytokines (IL-1β, IL-6, and TNF-α) in coxsackievirus B3 (CVB3)-infected mice, thus preventing viral myocarditis (Xu M. et al., 2020b).

Cytokine is a large group of molecules that are involved in the signaling process between cells (Roitt et al., 2001). Cytokines perform their function through the interaction with cytokine receptors that can be grouped in several distinct families. They are known to be crucial to innate and adaptive immunity, inflammation, cell growth, and differentiation (Spelman et al., 2006). Proinflammatory cytokines are produced predominantly by activated macrophages and are involved in the upregulation of inflammatory reactions. Targeting pro-inflammatory cytokine release has recently been regarded as a promising and attractive strategy to discover new drug leads. Currently, several studies report the activity of lupeol on pro-inflammatory cytokines.

A previous study demonstrated that lupeol at concentrations of 50 μM and 100 μM strongly reduced IL-8 release from the human colon epithelial cell line COLO 205. In addition, the secretion of IL-6, IL-12, and TNF-α from LPS-induced RAW 264.7 cells was prevented by the pretreatment with 10 μM, 50 μM, and 100 μM of lupeol (Lee et al., 2015). The result was supported by the previous study by Zhu et al. (2016) which revealed the ability of lupeol to decrease the production of IL-12, IL6, IL-1β, and TNFα from M1 activated macrophages. Lupeol also inhibited the expressions of pro-inflammatory cytokines, thus preventing TNF-α/IFN-γ-stimulated keratinocyte stimulation by blocking the signaling molecules, including transducer and activator of transcription 1, mitogen-activated protein kinases (p38 and ERK), and nuclear factor-κB in HaCaT cells (Bae et al., 2023). The mice’s ear oedema induced by 12-O-tetradecanoyl-phorbol acetate (TPA) was also suppressed by the topical treatment of lupeol (isolated from *Pimenta racemosa*) at 0.5 and 1 mg/ear. The anti-inflammatory effect was confirmed by the inhibition of myeloperoxidase activity in TPA-treated ears. However, lupeol had less anti-inflammatory effect on arachidonic acid-induced mice. The treatment of lupeol in lipopolysaccharide-stimulated mice peritoneal macrophages showed significant inhibition on the cytokine (TNFα, IL-1β) and PGE2 production (Fernández et al., 2001).

The anti-inflammatory effect of lupeol has also been reported to prevent atherosclerotic plaque formation. A previous study by Saha et al. (2020) reported the ability of lupeol to counteract the proinflammatory signaling triggered by a major product of oxidative stress-mediated cholesterol oxidation (7-keto-cholesterol). According to the study, lupeol pretreatment significantly raised ROS. Notably, autophagy induction requires both mitochondria and ROS. Through the promotion of proinflammatory M1 macrophage polarization, the compromised autophagy of macrophages enhances the immunological response. Treatment with lupeol of M1 monocyte derived macrophages M (IFN-γ/LPS) was able to downregulate IL-12. Moreover, pretreatment of macrophages with lupeol inhibited IL-12 and IL-1β release. A lupeol derivative chemical triggers autophagy, which results in cell death. Research revealed that cellular lipid accumulation and 7 KC-mediated cell death are both decreased by autophagy induction.

Beserra et al. (2019) also reported the importance of lupeol on modulating inflammation associated with diabetic conditions. Lupeol was found to be able to modulate inflammation, thus enhancing wound healing in streptozotocin-induced hyperglycemic rats. Immunohistochemical analyses showed decreased intensity of NF-κB and increased intensity of FGF-2, TGF-β1, and collagen III. Moreover, lupeol suppressed IL-6 levels and enhanced IL-10 levels (Beserra et al., 2019). This result was supported by a previous study that reported the ability of lupeol to reduce TNF-α and IL-6 levels in adipose tissue of type-2 diabetic rats (Daniel, 2023). Topical application of lupeol also improves skin wound healing in rats by reducing n in proinflammatory cytokines (TNF-a, IL-1β, and IL-6) (Beserra et al., 2020).

The anti-inflammatory effect of lupeol also contributes to the prevention of acute liver injury. Lupeol reduced the expression of TNF-α in lipopolysaccharide (LPS)/D-galactosamine (D-GalN)-induced mice liver injury (Huang et al., 2021). Lupeol effectively suppresses cholangiocarcinoma growth by its anti-inflammatory effect on TNF-α expression level, while showing no effect on IL-6 and CXCL-8 in human umbilical vein endothelial cells (HUVECs) (Kangsamaksin et al., 2017).

Lupeol acetate isolated from a plant latex (*Himatanthus drasticusis*) revealed anti-inflammatory activities by inhibiting neutrophil migration to the peritoneal cavity, reducing iNOS expression, as well as preventing dextran- and carrageenan-induced paw edema in mice. Moreover, it showed a dose-dependent inhibition on myeloperoxidase (MPO) release from human neutrophils (Lucetti et al., 2010). Oral administration of lupeol fraction (LF) of *Crateva adansonii* at 100 mg/kg revealed a significant inhibition on cytokine levels of carrageenan-induced rats, these include TNFα, IL-6, IFN γ, IL-1α, macrophage inflammatory protein (MIP), monocyte chemoattractant protein-1 (MCP-1), and regulated upon activation normal T cells expressed and presumably secreted (RANTES) (Rathinavel et al., 2021). Moreover, the treatment of lupeol isolated from *Diplotropis ferruginea* Benth in ovalbumin immunized-BALB/c mice caused a reduction of IL-4, IL-5, and IL-13, which was comparable to those of dexamethasone-treated mice (Vasconcelos et al., 2008). Lupeol from *Salvia willeana* was able to reduce the levels of IL-1β by 62% in ear homogenates of mice (Vonaparti et al., 2008).

Currently, a series of heterocyclic derivatives, including pyrazines along with oximes and indoles, which were synthesized from lupeol, have been found to inhibit the release of TNF-α and IL-1β from both RAW 264.7 and J774A.1 cells (Bhandari et al., 2014). There is a startling rise in immune system disorders linked to chronic inflammation in modern human culture. Recently, inflammatory processes have been connected to numerous additional illnesses, including cancer (Hrdý et al., 2020; Greten and Grivennikov, 2019). The specific inhibition of Ras farnesylation by Ras-farnesyltransferase and the irreversible suppression of cytosolic thioredoxin reductase TrxR-1, which results in an excess of reactive oxygen species, or ROS, are the basis for the anti-cancer (pro-apoptotic) action. The inhibitory effects of caspase 1, which is required for the ultimate processing of two important proinflammatory cytokines, IL-1β, and IL-18, and the inhibition of the IKK kinase β subunit, which disrupts the function of NF-κB, the central regulator of immune response, and affects the expression of pro-inflammatory genes regulated by this factor, are the sources of the anti-inflammatory properties (Bernier, 2006).

Lupeol was found to be able to switch macrophage phenotypes from M1 to M2 by decreasing the expression of CD86 (a typical M1 macrophage marker) while increasing the expression of CD206 (a typical M2 macrophage marker). Besides, IRF5, a transcription factor that is critically involved in M1 polarization, was downregulated in M1 macrophages after being incubated with lupeol, associated with a marked decrease in the phosphorylation of p38 mitogen-activated protein kinase (Zhu et al., 2016). The result was in accordance with another previous study, which revealed the ability of lupeol to inhibit M1 macrophage polarization while elevated M2 macrophage polarization (Li J. et al., 2021b). Lupeol was found to inhibit the expression of pyroptosis-associated proteins in macrophages during experimental autoimmune myocarditis (EAM). In addition, lupeol decreased pyroptosis in both bone marrow-derived macrophages (BMDMs) and THP-1-derived macrophages by *in vitro* studies. Lupeol also reduced the M1 polarization of macrophages both *in vivo* and *in vitro* (Xiong et al., 2024). Lupeol inhibited the activation of astrocytes/microglia that are associated with pathological processes following traumatic brain injury in mice. The immunoblot analysis showed a reduction of expression of GFAP. The result was supported by immunofluorescence analysis, which revealed the reduction of immunofluorescence reactivity of GFAP and Iba-1 (Ahmad et al., 2022). Lupeol also reduced the gene expression and protein secretion of T helper (Th) 2 cytokines, Th1 cytokines, and pro-inflammatory cytokines in ear tissue, as well as immunoglobulin (Ig) E (total and DFE-specific) and IgG2a levels in serum. The treatment of lupeol at 1 and 10 mg/kg significantly reduced ear thickness in atopic dermatitis-induced mice. The epidermal and dermal thickening and immune cell infiltration in ear tissue were also prevented after lupeol treatment (Bae et al., 2023).

Moreover, the treatment of lupeol isolated from *D. ferruginea* Benth. in ovalbumin immunized-BALB/c mice caused a reduction of cellularity and eosinophils in the bronchoalveolar lavage fluid (Vasconcelos et al., 2008). Lupeol from *S. willeana* showed inhibition on T-cell proliferation, which was obtained from human blood, in a concentration dependent manner 72 h after phytohemagglutinin (PHA) stimulation (Vonaparti et al., 2008). In contrast, lupeol isolated from *Hylocereus polyrhizus* at concentrations ranging from 6.25 to 100 μg/mL showed stimulation on the proliferation of lymphocytes obtained from mice spleen (Wahdaningsih et al., 2021). Lupeol isolated from *Crataeva religiosa* has been shown to suppress phagocytic function by *in vitro* and *in vivo* studies. In addition, it decreased delayed type hypersensitivity response in mice, particularly at 100 and 200 mg/kg p.o. CD4^+^ and CD8^+^ T cell count was also reduced after treatment with lupeol (Bani et al., 2006). In contrast, previous studies reported the ability of lupeol (isolated from *H. polyrhizus*) to enhance macrophage phagocytosis of latex beads (Wahdaningsih et al., 2020) and delayed-type hypersensitivity responses in mice against *Leishmania donovani*, thus decreasing the splenic parasite burden (Kaur et al., 2019).

Through modulating the expression of IL-2, IL-4, IL-5, ILβ, proteases, α-glucosidase, cFLIP, and NFκB, lupeol has cytostatic effects on cancer cells (Gunasekaran et al., 2022). Also significantly raises the rate at which cancer cells express BCL-2, BAX, caspases, and the PI3K-AKT-mTOR signaling pathway (He et al., 2018; Prasad et al., 2018).

According to the literature reported by Maurya et al. (2012), the results of QSAR analysis reveal that lupeol (7, triterpenoids-2) has immunomodulatory action and anti-inflammatory activity due to its high binding affinity to human receptors viz., NFκB P52, tumor necrosis factor (TNF-α), nuclear factor NFκB P50, and cyclooxygenase-2.

The structure-activity relationship (SAR) states that the methyl group’s varied location at ring E may be the cause of their actions. Pentacyclic triterpenes may be inhibited by the substitution of one methyl group on C-19 and the removal of one methyl group on C-20. The pentacyclic triterpenes’ different methyl group (ring E) positions may play a role in their contrast-enhancing properties. though the connection between their activities and chemical structure is still unknown, it is noteworthy that the presence of methanol at position C-17 and the substitution of one methyl group at position C-20 (ring E) may be crucial for the inhibition effect on the production of pro-inflammatory cytokines (Harun et al., 2020). In general, the presence of an oxygenated group at C-3 and a carboxyl group at C-28 of ring A may enhance its immunosuppressive activity on ROS production and human neutrophil chemotaxis, based on chemical structure and immunomodulatory effect analysis (Mawa et al., 2016).

### 5.6 Analgesic and anxiolytic properties

Analgesics are medications that, without substantially changing awareness, selectively reduce pain by affecting the central nervous system or peripheral pain mechanisms (Deshmukh et al., 2014). As an unpleasant feeling and an emotional experience connected to actual or prospective tissue damage, pain is thought to be an instinctive behavior unique to humans. Its function is to notify the body’s defense systems so they can respond to the stimulus and prevent additional tissue damage (Yam et al., 2018). According to Yam et al. (2018), there are three primary categories of pain: inflammatory, neuropathic, and nociceptive pain. Both *in vitro* and *in vivo*, lupeol has a variety of pharmacological actions, including the capacity to be both analgesic and antinociceptive (Rathinavel et al., 2021; De Lima et al., 2013).

Lupeol and common medications in the Randall-Selitto test considerably prolonged the rats’ reaction, which decreased the elicited pain. Lupeol’s ability to raise the threshold for intact paws implies that both peripherally and centrally mediated activities may have analgesic effects. The biphasic formalin test assesses pain originating from the nervous system (first phase) and the inflammatory system (second phase) (Chen et al., 2012). Because it is associated with tissue injury, this test is suggested as fundamental pain research to investigate the mechanism of analgesic medicines. Significant analgesic action was demonstrated by lupeol in both phases, with 60% inhibition in the first phase and 31% in the second. According to De Lima et al. (2013), the antinociceptive effect operates in the second phase. Central sensitization develops as a result of increased afferent input into the spinal cord’s dorsal horn brought on by inflammatory pain. At the site of tissue injury, a variety of mediators are generated, including prostaglandins and nitric oxide (NO). Lupeol reduces pain and edema in a formalin test paradigm by inducing inflammatory mediators (Adzu et al., 2015). These chemical inflammatory mediators are created from necrotic tissue during the inflammatory process, and they interact to activate nociceptors in the inflammatory area (Yam et al., 2018). In lipopolysaccharide-stimulated RAW 264.7 macrophage cells, lupeol acetate dose-dependently suppresses the generation of nitric oxide (NO) (Chen et al., 2012).

According to De Lima et al. (2013) and Rathinavel et al. (2021), the primary mechanism by which lupeol exerts its activity is through the suppression of tissue responses to nociception-induced factors, primarily through the involvement of cytokines. Lupeol suppresses the elevated cytokine production, specifically IL-1β and TNF-α, that is locally caused by carrageenan. Tumor necrosis factor α (TNFα) and interleukin 1β (IL-1β) are key players in the potent proinflammatory actions that result in hyperalgesia (Yam et al., 2018). Lupeol was found to dramatically lower the production of TNF-α and IL-1β from lipopolysaccharide-stimulated macrophages when evaluated *in vitro*, which is consistent with the current data (De Lima et al., 2013; Rathinavel et al., 2021).

Anxiety is a future-focused mood state linked to getting ready for potential bad things that might happen in the future (Craske et al., 2011). Concern is seen as hard to regulate in clinical presentations like generalized anxiety disorder and is linked to a range of physical symptoms like weariness, restlessness, irritability, or sleep difficulties (Clayton and Karazsia, 2020). The finding that psychological stress, specifically anxiety and depression in humans, can trigger the release of pro-inflammatory cytokines like TNF-α has significant consequences for the pathology of humans (Maes et al., 1998; de Heer et al., 2014). Lupeol significantly reduces TNF-α production from lipopolysaccharide-stimulated macrophages, thereby reducing anxiety and depression (De Lima et al., 2013; Rathinavel et al., 2021).

### 5.7 Dermatoprotective actions

The primary function of the skin barrier is to keep the body safe from dangerous environmental conditions. The loss of skin integrity triggers a series of mechanisms that restore all epidermal defensive functions. Heat, radiation, chemicals, and a variety of other environmental elements can all harm the skin barrier. Any break in the skin barrier integrity exposes the organism to hazardous environmental elements (Malinowska et al., 2021). Lupeol is a pentacyclic triterpenoid with a hydroxyl group and an olefinic moiety, which contributes to its amphiphilic nature. This structure allows it to interact with cell membranes and exert protective effects against skin damage (Sharma et al., 2020).

Baserra et al. conducted an *in vitro* migration assay to examine lupeol’s impact on wound healing, focusing on proliferation, migration, and cell contraction, and to propose a potential mechanism of action. Lupeol was extracted from *Bowdichia virgilioides* using a 95% alcohol solution and was extensively purified. High doses of lupeol were found to decrease cell proliferation in fibroblasts and keratinocytes while maintaining cell viability. Additionally, lupeol enhanced wound healing in keratinocytes and promoted dermal fibroblast contraction in a collagen gel matrix (Beserra et al., 2018). The following year, Baserra et al. performed an *in vivo* study on rats, using the same extract from *B. virgilioides*, to investigate lupeol’s healing properties on wounds caused by streptozotocin-induced hyperglycemia in an excision wound model (Beserra et al., 2019).

Topical application of lupeol combined with cyclodextrin in SKH1 mice with UVB and DMBA-induced skin carcinoma demonstrated a reduction in tumor mass, inflammation, irritation, and skin degradation by enhancing skin physiological parameters such as TEWL, erythema, skin hydration, and sebum content (Minda et al., 2015). Treatment with lupeol (0.2% w/w) positively impacted all three stages of skin wound healing It exhibited anti-inflammatory properties by lowering levels of TNF-α, IL-1β, and IL-6 while increasing IL-10 cytokines. During the proliferative stage, it promoted angiogenesis and the expression of Ki-67, VEGF, EGF, and TGF-β1. In the tissue regeneration phase, it facilitated collagen fiber deposition (Beserra et al., 2020).

**FIGURE 5:**
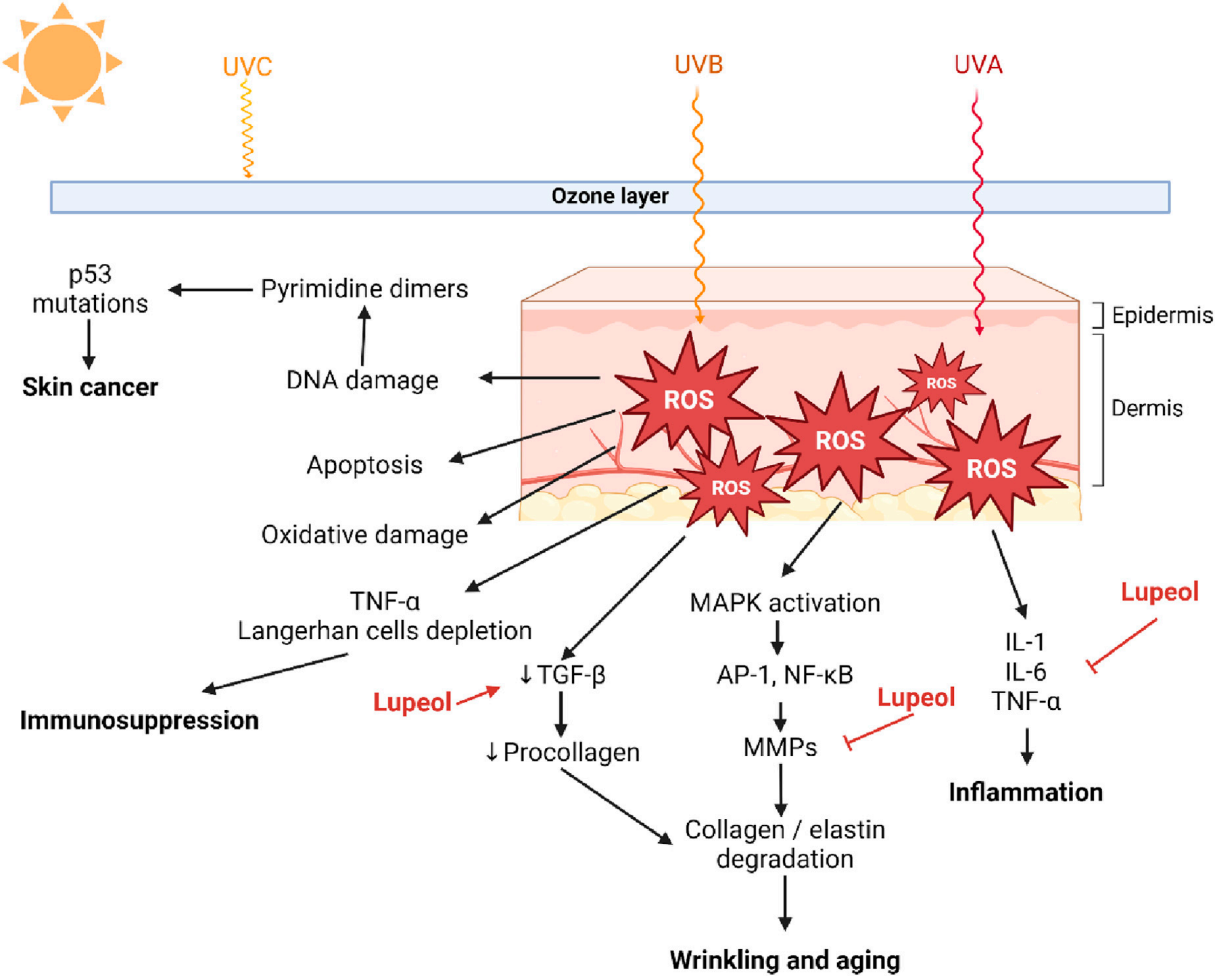
Mechanism of Lupeol’s Dermatoprotective actions.

Lupeol prevents senescence by inhibiting MMP-1, -2, -3, along with p-p53, p21, and p16 expression, and reducing SA-β-gal activity in FB models repeatedly exposed to UVA radiation. This indicates that lupeol could be an effective anti-aging agent (Park and Park, 2019). The mechanism of lupeol’s dermatoprotective actions can be seen on Figure 5.

### 5.8 Hepatoprotective and renoprotective potentials

The liver and kidneys play critical roles in the body’s waste removal mechanisms. Damage to these organs can lead to metabolic dysfunction, toxin buildup, and tissue atrophy (Baravalia et al., 2011). The kidneys filter waste products from the blood, maintain electrolyte balance, control erythropoietin secretion, and regulate blood pressure (Francois et al., 2007; Breyer et al., 2001). They also regulate vascular tone and sodium levels by secreting prostaglandins, which help to balance the renin-angiotensin system. Chronic kidney and liver damage can result from significant cellular injury caused by the excessive generation of reactive oxygen species (ROS) and oxidative stress, which activate inflammatory responses. These responses release inflammatory mediators such as NF-κB, TNF-α, NO, and IL-6 (Jena, 2012; Lu et al., 2008).

Renal injury, a condition caused by an abrupt, transient interruption of blood supply to the kidney followed by reperfusion, leads to glomerular and severe tubular damage, resulting in oxidative stress and apoptosis (Li et al., 2017; Hong et al., 2017; Singh and Chopra, 2004). The reperfusion process generates an excess of ROS, causing oxidative stress and promoting apoptosis and cell death, which result in kidney tissue destruction. Endothelial damage, leukocyte infiltration, and the production of inflammatory mediators further exacerbate the injury. This condition is a common complication following renal surgeries, including partial nephrectomy, kidney transplantation, and renal artery angioplasty (Malek and Nematbakhsh, 2015). However, there are no specific therapeutic drugs available for managing renal injury in clinical settings, highlighting the urgent need for effective treatments (Peng et al., 2023).

**FIGURE 6:**
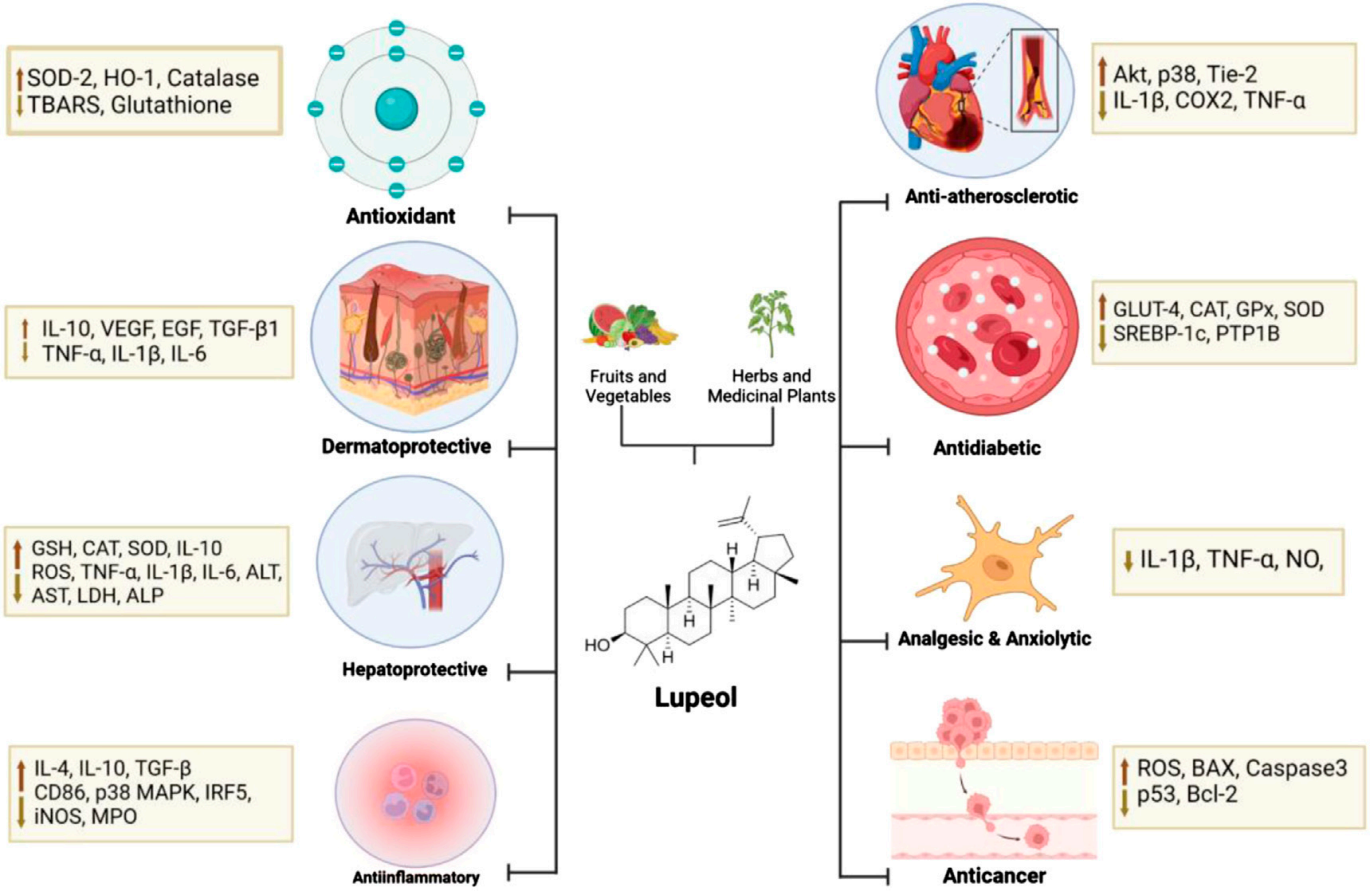
Impact of lupeol on various types of disorders.

Lupeol has shown significant potential as a hepatoprotective and renoprotective agent, making it a promising candidate for managing chronic liver and kidney diseases. The presence of a hydroxyl group (–OH) at the C3 position is crucial. This hydroxyl group is involved in hydrogen bonding and can contribute to the compound’s ability to interact with biological molecules, potentially influencing its hepatoprotective effects. The hydroxyl group can participate in hydrogen bonding, which is essential for interacting with biological molecules. This interaction can help lupeol modulate cellular pathways and protect liver cells from damage (Saleem, 2009; Panja et al., 2021). For instance, serum levels of ALT, AST, LDH, and ALP are commonly used to diagnose liver damage, as these enzymes are released into the bloodstream during hepatic injury. Preetha et al. (2006) found that lupeol can reduce the levels of these enzymes and also provide antioxidant effects, thereby protecting liver function. Additionally, Tiwari et al. (2019) found that lupeol has renoprotective activities by enhancing the activity of antioxidant enzymes such as glutathione (GSH), catalase (CAT), and superoxide dismutase (SOD), which reduces oxidative stress and protects kidney function. More evidence of lupeol’s hepatoprotective and renoprotective activities can be seen in Tables 4, 5. Lupeol demonstrates significant hepatoprotective and renoprotective properties through its antioxidant, anti-inflammatory, and anti-apoptotic mechanisms. These effects make it a promising candidate for managing chronic liver and kidney diseases. Future research should focus on large-scale clinical trials and novel delivery systems to enhance its therapeutic potential. The impact of lupeol on various types of disorders can be seen on Figure 6.

## 6 Challenges in therapeutic utilization of lupeol

Although lupeol possesses a diverse array of pharmacological effects, it has not yet been developed into a medicinal medicine. There are several problems, such as the inability to dissolve in water, restricted ability to be absorbed by the body, and a short amount of time it stays in the bloodstream (Siddique and Saleem, 2011). The oral bioavailability of lupeol is limited due to its high lipophilicity and low water solubility, as stated by Wang et al. (2016). In a study conducted by Khatal and Moore. (2019), it was discovered that the oral bioavailability of lupeol is less than 1%. Additional physicochemical characteristics that influence absorption include the dissolution rate of the substance, its apparent solubility in the gastrointestinal (GI) tract, its permeability, and its stability in various regions of the GI tract (Hörter and Dressman, 2001). However, it is crucial to adopt strategies that improve the capacity of lupeol to dissolve and the oral bioavailability (Park et al., 2023).

Currently, researchers are in the process of developing an improved version of lupeol to address these issues. Zhang et al. (2019) created a new nanosystem called lupeol-loaded PEGylated liposomes, which possess an appropriate shape, particle size, and encapsulation ratio. The incorporation of lupeol into PEGylated liposomes has effectively addressed the issue of its limited solubility in water and low absorption into the body. Priyanka et al. (2017) developed a solid lipid nanoparticle (SLN) to address these issues. Solid lipid nanoparticles (SLN) are widely recognized for their capacity to improve the bioavailability of medications with low solubility in water through uptake by the lymphatic system (Singh et al., 2013). The lupeol-containing extract was successfully generated by adjusting the type and concentration of lipids in solid lipid nanoparticles (SLN). The pharmacokinetic investigations of SLN-loaded extract showed a significant enhancement in the pharmacokinetic characteristics of lupeol when formulated in SLN form. This study presents the hitherto unreported pharmacokinetic properties of lupeol in the extract, as well as its improved bioavailability achieved through the use of SLN formulation.

In recent studies, researchers have explored the use of poly(lactide-co-glycolide) (PLGA) nanoparticles as a potential alternative. PLGA nanoparticles have several advantages, such as being biodegradable and non-toxic, and their degradation products are harmless lactic and glycolic acid (Panyam et al., 2002; Kim et al., 2006). Cháirez-Ramírez et al. (2015) developed poly(lactide-co-glycolide) (PLGA) nanoparticles containing lupeol and modified them with NF-κB. This improved formulation demonstrated enhanced bioavailability within the cell interior and exhibited the most potent anti-inflammatory effects at the greatest dosage of pure lupeol. This confirms that providing lupeol in an encapsulated form to the cell interior can boost the bioavailability of lupeol.

Multiple researchers have facilitated the development and assessed the effectiveness of nanoemulsions as a method for orally delivering lupeol, a pentacyclic triterpene. Nanoemulsion is a highly promising technique that enhances the oral bioavailability of medications with low solubility. Thus, the formulation procedure was implemented based on specific treatment needs and the chosen method of administration (Anton et al., 2008). A self-emulsifying drug delivery method, commonly referred to as a preconcentrated nanoemulsion (NP), can enhance the capacity of medications to dissolve in water and consequently enhance the ability of lipophilic pharmaceuticals to be absorbed by the body. The process of formulating nanoemulsion preconcentrate is uncomplicated and economical. It can also be utilized to enhance the ability of lupeol to be absorbed orally (Jyotshna et al., 2020).

Despite the notable advancements in formulation strategies aimed at enhancing the bioavailability of lupeol, it is crucial to evaluate their efficacy through clinical trials. Numerous recent clinical trials have been conducted to assess the efficacy of enhanced lupeol formulations in humans. Successfully executed clinical trials demonstrate promising results in treatment. Nonetheless, modifications to the dosage, concentration, and administration frequency are necessary to optimize the efficacy of lupeol in accordance with clinical standards. Moreover, additional clinical trials are required to evaluate the efficacy of lupeol as both a monotherapy and in conjunction with current treatments (Siddique and Saleem, 2011).

Recent clinical trials have demonstrated promising outcomes regarding the use of lupeol as a chemopreventive drug, indicating its potential to diminish the risk of cancer in future studies and serve as an alternative outcome in clinical research (Chaturvedi et al., 2008). The idea of chemoprevention utilizing lupeol remains nascent, with numerous obstacles to surmount. This includes establishing the appropriate dosage, ideal time and duration of exposure, and the specificity of cell types responsive to lupeol. Furthermore, its relative bioavailability and possible adverse effects or undesirable interactions must also be taken into consideration. The interaction of lupeol with dietary constituents necessitates further investigation and importance.

## 7 Toxicity and safety profile of lupeol

Lupeol has attracted much attention because of its low toxicity and wide pharmacological effects. According to Patocka and Patočka (2003), lupeol has been found to have no harmful effects in animal experiments, as mentioned in the sources. Lupeol, when evaluated at doses ranging from 40 to 200 mg/kg using different treatment procedures (long or short-term), did not exhibit any adverse effects on the overall health of animals. A study conducted by Saleem (2009) found that mice who were given lupeol through intraperitoneal administration at a dosage of 40 mg/kg did not exhibit any signs of toxicity or mortality. Furthermore, another discovery revealed that lupeol, when evaluated at doses ranging from 40 to 200 mg/kg using different treatment regimens (long or short-term), did not exhibit any adverse effects on the overall health of animals (Saleem, 2009). The oral administration of lupeol at a dose of 100 mg/kg for a duration of 7 days did not result in any deaths or systemic toxicity in mice (Preetha et al., 2006; Zhu et al., 2016).

In addition, Kwon et al. (2015) conducted a study that evaluated the potential toxicity of lupeol by examining its impact on the viability of HaCaT keratinocytes and SEB-1 sebocytes. They showed that lupeol treatment did not cause cell death under the same conditions as prior cellular investigations (concentration range of 0–20 mM for 24 h of incubation) in either kind of cell. Ruiz-Rodríguez et al. (2017) used an *in silico* analysis to provide predictions about the toxicological safety of lupeol. The findings indicated that lupeol and its analogues have low toxicity and do not strongly bind to nuclear receptors. Hence, this aspect should be taken into account when guiding future investigations into the utilization of lupeol and its analogues as prospective pharmaceuticals for disease treatment. In a study investigating the effectiveness of lupeol in preventing the growth of blood vessels and its potential as an antiangiogenic drug, mice with Ehrlich ascites carcinoma (EAC) and Dalton’s lymphoma ascites (DL) tumors were given a dose of 40 mg/kg body weight of lupeol through intraperitoneal injection after the tumors had developed. The results showed that this treatment did not have any toxic effects (Vijay Avin et al., 2014). Collectively, these observational studies have consistently demonstrated that the use of lupeol does not pose any significant health risks. Moreover, these findings have the potential to provide a new avenue for exploring the use of lupeol in clinical trials.

## 8 Technological advances in lupeol enhancement

Recent advancements in lupeol enhancement technologies have primarily focused on addressing its inherent challenges, such as low solubility, poor bioavailability, and limited stability, which have restricted its therapeutic potential. Lupeol, recognized for its powerful anti-inflammatory, antioxidant, and anticancer properties, often faces reduced efficacy due to these limitations. Nevertheless, the advancement of innovative transportation systems, such as carriers based on nanotechnology, methods of encapsulation, and chemical changes, has greatly enhanced the pharmacokinetic characteristics of lupeol. These advancements provide more precise regulation of the release, improved absorption into the body, and specific administration, hence increasing the efficacy of lupeol for a range of medical uses (Ramos-Hernández et al., 2018; Tian et al., 2024).

Research study by Pârvănescu et al. (2021) demonstrated the effective integration of Lupeol into an oleogel with favorable physicochemical properties, making it suitable for topical application. *Ex vivo* studies confirmed that lupeol effectively penetrates the skin and remains within skin layers, thereby ensuring localized therapeutic effects. The formulation’s biocompatibility *in vitro* tests demonstrated its safety for human skin cells and its ability to reduce inflammation and promote skin regeneration.

Nanotechnology-based medications are specifically designed to minimize toxicity and enhance health results. In addition, solid nanoparticles offer significant benefits in drug development due to their biophysical stability and the capacity to change the formulation of pharmaceuticals to enable controlled drug release (Sahu et al., 2021). Nanoparticles can affect drug target site efficacy. Despite being biologically active *in vitro*, small molecular medicines have poor pharmacokinetic and pharmacodynamic characteristics. Nanoparticles tune their physicochemical qualities to control these factors (Sindhwani and Chan, 2021). Although lupeol has a diverse array of pharmacological effects, it has not yet been developed into a medicinal medicine. Several challenges exist with this medicine, including its low solubility in water, limited bioavailability, and short plasma half-life. To enhance its effectiveness, a new drug carrier is needed (Zhang et al., 2019). Three constitutive processes determine oral bioavailability: bioaccessibility, transport across the intestinal epithelium, and metabolism. Oral bioaccessibility can be increased by emulsions, self-dispersing lipid formulations, lipid carriers, nano-emulsions, solid lipid nanoparticles, polymeric nanoparticles, etc. (Rocha-Guzmán et al., 2021).

Numerous studies are currently being conducted to investigate the potential of nanotechnology to improve the bioavailability of lupeol. The research by Cháirez-Ramírez et al. (2015) shows that PLGA nanoparticles loaded with lupeol in lower doses work just as well as higher doses of pure lupeol on anti-inflammatory effects. The polymer poly (D,L–lactide) (PLA), either alone or in combination with copolymers such as glycolide, has been extensively employed for the formulation of low water solubility payload particles. It has been reported to be highly effective in enhancing the release rate (Cháirez-Ramírez et al., 2015). Lipid nanoparticles may penetrate biological barriers, optimize medication efficacy, and release drugs at targeted locations with lower toxicities than free drugs. Because macrophages naturally phagocytose these nanoparticles without surface alteration, these nanocarriers allow passive targeting. Solid lipid nanoparticles (SLN) are widely recognized for their capacity to improve the bioavailability of medicines with low water solubility through lymphatic uptake (Jesus et al., 2023; Priyanka et al., 2017). According to Priyanka et al. (2017) and Zhang et al. (2019), the use of solid lipid nanoparticles (SLN) loaded with lupeol and PEGylated liposomes loaded with lupeol results in an improvement in the bioavailability of the compound and solves its poor hydrophilicity. There are numerous approaches to using nanoformulation to increase the bioavailability and solubility of lupeol, although research in this area remains ongoing.

Lupeol contains a bioactive lupane-type structure that is highly useful. It has numerous natural stereogenic centers and may be easily modified at positions C-3 and C-20. Therefore, certain variations of lupeol were obtained from plants or artificially created with the purpose of enhancing its solubility in water, absorption, distribution, metabolism, excretion (ADME), bioavailability, and efficacy (Tsai et al., 2016). The synthesis of lupeol can be achieved through a complex series of reactions involving Grignard reagent coupling, TBAF treatment, catalytic hydrogenation, followed by methylation, silylation, alkylation, reduction, and ultimately cyclization (Surendra and Corey, 2009).

The derivatives that were generated can be classified into six distinct groups: indole derivatives, 3-keto lupeol, 3-ester derivatives, 3-oxime, pyrazine derivatives, sulfur derivatives, and allylic oxidation, which is an aldehyde (Bhandari et al., 2014). An esterification method was used to get the lupeol derivatives. Lupeol esters were synthesized using suitable carboxylic acids or carboxylic acid anhydrides as acylating agents. The specific esters obtained were lupeol acetate, lupeol propionate, lupeol isonicotinate, lupeol succinate, and lupeol acetylsalicylate (Malinowska et al., 2019; Tsai et al., 2016). According to a study by Malinowska et al. (2019), lupeol acetate, isonicotinate, and propionate were the best compounds for increasing the growth of human skin cells compared to control samples. This resulted in an increase in cell concentration that was greater than thirty percent. In comparison to lupeol, lupeol acetate (LA), a derivative of lupeol, exhibits superior bioavailability and reduces inflammation in mice produced by carrageenan (Wang et al., 2016). Lupeol analogues, including 30-formyl lupeol, lupeol acetate, and lupeol esterification with other compounds, exhibit notable effects in promoting glucose uptake in skeletal muscle cells. This suggests that they have the potential to lower blood glucose levels by enhancing the utilization of glucose through skeletal muscles (Khan et al., 2014b). The chemical alteration of lupeol has the potential to not only affect the biological activity of the derivative that is generated but also to improve its effectiveness and bioavailability.

## 9 Future perspectives and directions

Lupeol has demonstrated diverse pharmacological actions in both laboratory and living organism settings. These benefits encompass its anti-inflammatory, anticancer, anti-arthritis, antidiabetic, cardioprotective, nephroprotective, and hepatoprotective properties. The low solubility and bioavailability of lupeol have resulted in a lack of clinical examinations (Liu K. et al., 2021a). Lupeol is currently in development with formulations that enhance the bioavailability of the compound (Sahu et al., 2021). One such approach involves the utilization of nanotechnology, including lipid carriers, nanoparticles, and other techniques. Several studies mention that nanoformulation can enhance lupeol’s bioavailability and effectiveness. Additionally, modifying the structure of lupeol, such as through the introduction of ester groups, can further enhance its effects. For example, a study conducted by Wang et al. (2016) demonstrated that lupeol acetate, a derivative of lupeol modified with esterification, increased bioavailability and reduced inflammation in mice that is induced with carrageenan.

Numerous studies have shown that lupeol has enormous potential in this area of medicine. Research by Sudharsan et al. (2006) showed results that the addition of lupeol and its ester reduces oxidative stress and provides protection against CP-induced cardiotoxicity by directly eliminating free radicals, which can potentially regulate mitochondrial activity. Adverse drug reactions frequently disrupt cancer chemotherapy. These findings indicate that the effectiveness of CP treatment is enhanced when combined with lupeol and further improved when combined with lupeol linoleate. The fact that lupeol did not cause toxicity to normal cells and that it performed synergistically in combination treatments makes it a good candidate for use as an adjuvant to anticancer and anti-inflammatory medications that are currently being researched (Sharma and Gupta, 2022). Research should be undertaken to identify various conjugated proteins throughout treatments, with the aim of uncovering new targets and markers of therapeutic efficacy. Furthermore, it is imperative to do pharmacokinetic research on lupeol to improve its miscibility qualities, as well as its systemic bioavailability and absorption. As lupeol undergoes further development for main or adjunctive therapy and gets through clinical trials, it can be commercialized with the benefit of treating a wide range of diseases.

## 10 Conclusion

Scientists are becoming more interested in chemical compounds originating from natural sources, particularly substances obtained from plants, due to their numerous pharmacological characteristics that contribute to human health. Lupeol is a triterpene that is widespread across various species and can be found in a range of edible vegetables and fruits. Additionally, there are several analytical techniques for identifying lupeol in plant sources. From the above claimed information, we found that lupeol is a source of biologically active phytochemicals that can be used for the treatment of various types of disorders like diabetes, cardiovascular diseases, dermatological disorders, chronic liver and kidney disease, inflammation, and cancer.

However, despite its significant health potentials, the observed *in vivo* experiments have indicated that lupeol has limited bioavailability and poor water solubility. To overcome this obstacle, researchers have directed their efforts towards several ways that, in theory, could increase bioavailability and subsequently improve health advantages. Additionally, various lupeol derivatives have been created to overcome these limitations and further boost its bioactivity. This review offers information on the strategies employed to enhance the bioavailability of lupeol and address its limited solubility. Nano-based approaches such as solid lipid nanoparticles (SLN) and PLGA are some of the lupeol formulations with increased bioavailability. Based on a literature survey, it has been discovered that lupeol possesses a highly remarkable pharmacological profile. This suggests that lupeol could potentially be utilized as a medicinal treatment for various types of illnesses in the future. The purpose of this study is to provide researchers in both fundamental and practical research with a thorough understanding of lupeol, enhance the effective utilization of lupeol, and finally facilitate the development of lupeol as a novel pharmaceutical drug.
